# Unraveling the molecular Nexus of Alzheimer’s Disease and HIV encephalitis: The role of cellular senescence and transcriptional regulation

**DOI:** 10.1371/journal.pone.0355548

**Published:** 2026-08-13

**Authors:** Hao Zhang, WenJun Chen, ShuYou Yuan, ShaoXiang Ding, HongXia Bao, Bo Cai, JunKai Sun, Wei Lu, HaoGang Zhu, GuoXun Shi

**Affiliations:** 1 Geriatrics Center, Wuxi Second Geriatric Hospital, Wuxi, Jiangsu Province, China; 2 Neurology Department, Wuxi Second Geriatric Hospital, Wuxi, Jiangsu Province, China; 3 Laboratory Department, Wuxi Second Geriatric Hospital, Wuxi, Jiangsu Province, China; 4 Pathology department, Wuxi Second Geriatric Hospital, Wuxi, Jiangsu Province, China; 5 Department of Interventional Radiology, Wuxi No. 5 People’s Hospital, Wuxi, Jiangsu Province, China; 6 Rheumatology Department, Wuxi Second People’s Hospital, Wuxi, China; University of Texas Medical Branch at Galveston, UNITED STATES OF AMERICA

## Abstract

Alzheimer’s disease (AD) and HIV-associated neurocognitive disorder (HAND) share progressive cognitive decline. Their common molecular mechanisms remain poorly understood. Current therapeutic approaches lack effective biomarkers for early diagnosis and intervention. We integrated transcriptomic profiles from multiple independent cohorts across brain tissues and blood. We systematically evaluated diagnostic performance using machine learning algorithms including Random Forest, Support Vector Machine, and XGBoost. Notably, FOXO3 emerged as the top cross-disease biomarker. FOXO3 achieved diagnostic accuracy in AD temporal cortex (area under the curve [AUC] = 0.922, 95% CI: 0.885–0.959). FOXO3 showed diagnostic performance in HAND frontal cortex (AUC = 0.771, 95% CI: 0.724–0.818). FOXO3 expression positively correlated with APP (R = 0.558). FOXO3 expression negatively correlated with MAPT (R = −0.690) and SORL1 (R = −0.856). ACE showed strong positive correlation with FOXO3 (R = 0.685), suggesting vascular involvement. STAT3 and ZNF341 were identified from 21 transcription factor candidates. STAT3 ranked first in HAND integrated cohort (n = 107, AUC = 0.759). STAT3 may function as an inflammatory mediator through JAK-STAT signaling. ZNF341 showed strongest transcriptional association with disease status (β = 5.09 in AD, β = 4.85 in HAND). The two-gene panel (FOXO3-ZNF341) achieved diagnostic accuracy in blood samples (AUC = 0.764, n = 329). This performance approaches clinical utility thresholds. Blood-based detection offers non-invasive diagnostic potential. Saturation analysis identified three molecules as optimal panel size. Marginal AUC gains declined below 0.02 beyond this threshold. FOXO3, STAT3, and ZNF341 showed stable selection frequency (97%, 65%, and 100%, respectively). PI3K-Akt and FoxO signaling pathways were enriched, which are known to regulate apoptosis and cell survival. Taken together, these computational findings indicate FOXO3 transcriptional regulatory activity in neurodegeneration. The three-molecule panel represents candidate blood-based diagnostic biomarkers. These candidate biomarkers warrant further investigation in independent clinical cohorts.

## Introduction

Alzheimer’s disease (AD) represents the most common neurodegenerative disorder worldwide. AD affects over 50 million individuals globally and imposes substantial healthcare burdens. The disease manifests as progressive cognitive decline and functional impairment [1]. Pathological hallmarks include amyloid-β plaques and tau tangles. Current therapeutic approaches demonstrate limited efficacy in disease modification [2].

HIV-associated neurocognitive disorder (HAND) constitutes a distinct neurodegenerative condition affecting 30–50% of people living with HIV despite antiretroviral therapy (ART) [3]. Both AD and HAND share overlapping clinical features including progressive cognitive deterioration and quality of life decline [4]. Healthcare costs and caregiver burdens remain substantial in both conditions. These similarities suggest potential common molecular mechanisms underlying neurodegeneration [4].

Current diagnostic approaches rely heavily on clinical assessments. AD diagnosis often occurs after significant neuronal damage has accumulated [5]. HAND diagnosis depends on neuropsychological testing without validated molecular biomarkers [6]. Cellular senescence has emerged as a critical mechanism in neurodegeneration. Senescent cells accumulate in aging brains and secrete inflammatory factors. This senescence-associated secretory phenotype (SASP) promotes chronic inflammation [7]. Recent evidence implicates cellular senescence in both AD and HAND pathogenesis [8,9]. We hypothesized that senescence-related genes drive shared transcriptional alterations. These genes may serve as diagnostic biomarkers for both conditions.

Transcriptomic analyses combined with machine learning offer robust approaches for biomarker discovery. Studies identified dysregulated genes involved in inflammation and synaptic function in AD [10]. HAND transcriptomic studies showed immune activation and neuronal dysfunction [11]. However, direct comparisons between AD and HAND remain limited. Machine learning approaches handle high-dimensional genomic data effectively. Ensemble strategies combining multiple algorithms improve diagnostic accuracy. Multi-dataset integration addresses sample size limitations through batch effect correction. Such approaches have identified robust biomarkers in cancer and neurodegeneration. However, systematic comparisons of ensemble methods in AD and HAND remain unexplored [12].

This study employed comprehensive bioinformatics approaches to identify shared molecular signatures. We integrated multiple transcriptomic datasets from NCBI Gene Expression Omnibus (GEO). Machine learning models including Random Forest, Support Vector Machine, and eXtreme Gradient Boosting (XGBoost) identified key molecular markers. We systematically evaluated 129 ensemble algorithm combinations for optimal diagnostic performance. Transcription factor networks were reconstructed using regulatory inference. Our objective was to uncover shared mechanisms between AD and HAND. The findings may inform diagnostic and therapeutic development for both neurodegenerative conditions.

## Materials and methods

### Study design and data availability

This study analyzed publicly available de-identified transcriptomic datasets from NCBI GEO database. No ethical approval or patient consent was required as per institutional guidelines for secondary data analysis. All datasets are summarized in Table 1. For clarity, dataset information appears in both the summary and Methods sections: the summary provides an overview of dataset types and sources, while the Methods section details specific sample sizes, preprocessing steps, and analytical parameters for reproducibility.

**Table 1:** Comprehensive overview of Alzheimer’s and HIV-related neurodegenerative datasets.

GSE ID	Condition (N)	Age	Sex (M/F)	Diagnosis (per-group N)	Disease Stage	Brain Region/ Tissue
GSE3489	HAND (14) ^[†]^	52.3 ± 8.7^[§]^	N/S	HIVE+ (8), HIVE- (6)	N/S	Frontal cortex (BA46)
GSE28160	HAND (21)	N/S	N/S	ART+ HAND (7), ART- HAND (8), Ctrl (6)	N/S	Multiple brain regions
GSE35864	HAND (24) ^[‡]^	N/S	N/S	Ctrl (6), HIV (6), HAND (7), HIVE (5)	N/S	Basal ganglia, White matter, Frontal cortex
GSE37263	AD (16)	78.5 ± 9.2	N/S	AD (8), Ctrl (8)	N/S	Temporal cortex (BA22)
GSE48350	AD (84)	20–99 yrs^[¶]^	124M/ 129F	AD (28), Ctrl (56)	N/S	Hippocampus, Entorhinal cortex, Superior frontal gyrus, Post-central gyrus
GSE63060	AD (Blood) (329)	74.2 ± 7.8	191M/ 138F	AD (145) ^[**]^, MCI (80) ^[**]^, Ctrl (104) ^[**]^	AD/MCI vs. Ctrl	Blood
GSE122063	AD (31)	N/S	N/S	VaD (8), AD (12), Ctrl (11)	VaD vs. AD	Frontal cortex, Temporal cortex
GSE132903	AD (195)	N/S	N/S	AD (97), Ctrl (98)	AD vs. Ctrl	Middle temporal gyrus (BA21)

† GSE3489: 14 unique individuals (6 HIVE- + 8 HIVE+), each with A/B technical replicates = 28 arrays total.

‡ GSE35864: 24 subjects × 3 brain regions (basal ganglia, white matter, frontal cortex) = 72 samples total.

§ GSE3489 age (52.3 ± 8.7 yrs) derived from original manuscript Methods section; age/sex data for other HAND datasets not available in GEO records.

¶ GSE48350 spans an age range of 20–99 years, reflecting the heterogeneous donor population from the Harvard Brain Tissue Resource Center.

** GSE63060 per-group N from raw GEO data (145 AD, 80 MCI, 104 Control). The AddNeuroMed study final classification reported AD (165) and Ctrl (164) after diagnostic adjudication of MCI cases; see Methods for details.

Abbreviations: AD, Alzheimer’s Disease; HAND, HIV-Associated Neurocognitive Disorder; HIVE, HIV Encephalitis; ART, Antiretroviral Therapy; MCI, Mild Cognitive Impairment; VaD, Vascular Dementia; Ctrl, Control; N/S, Not Specified; BA, Brodmann Area.

**Fig 1:**
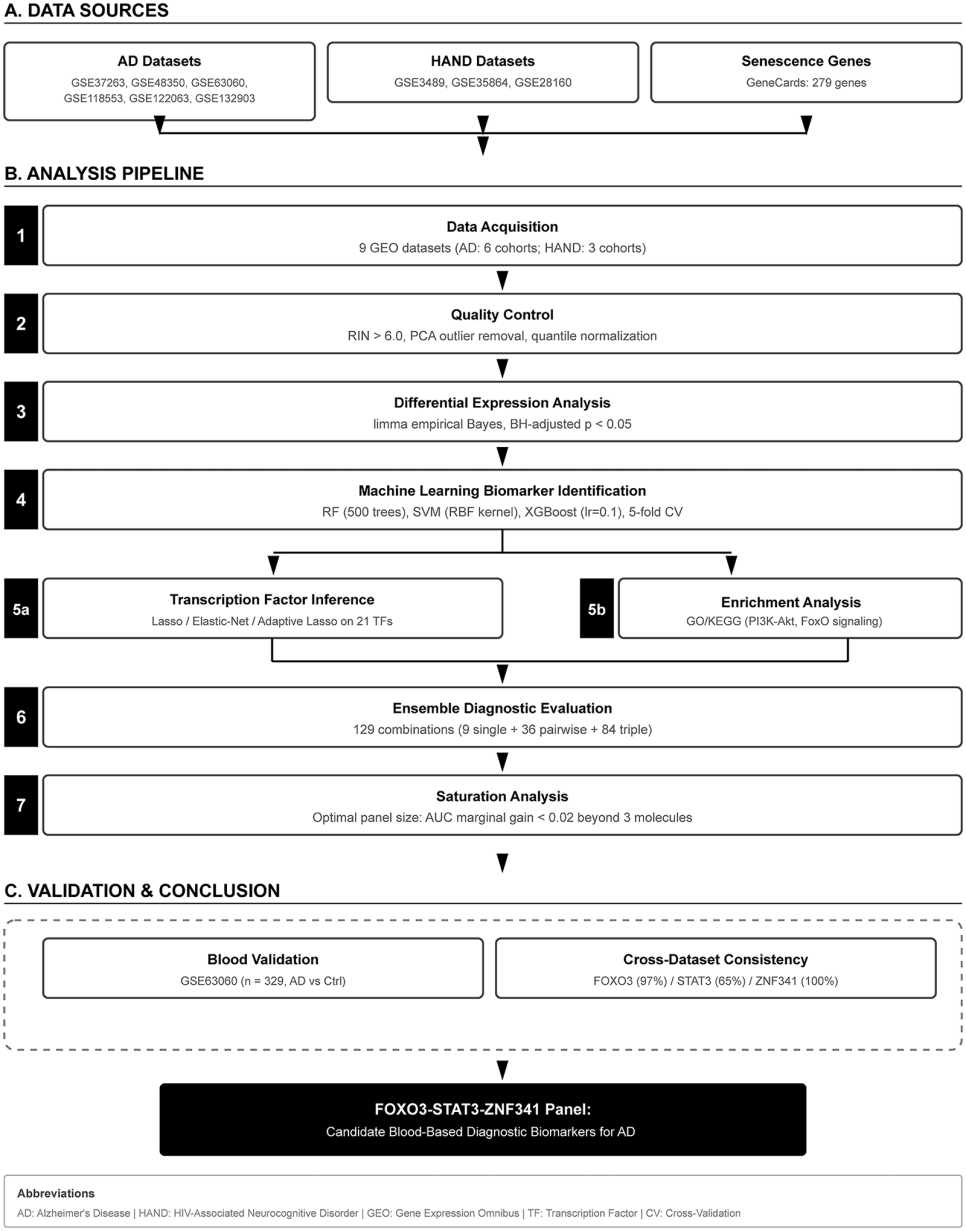
Study design and analytical workflow. The seven-step pipeline includes: (i) data acquisition from AD (GSE37263, GSE48350, GSE63060, GSE122063, GSE118553, GSE132903) and HAND (GSE3489, GSE35864, GSE28160) cohorts; (ii) quality control and batch effect correction; (iii) differential expression and enrichment analysis (limma, BH-adjusted p < 0.05); (iv) machine learning biomarker identification (RF, SVM, XGBoost, 5-fold CV); (v) transcription factor inference (Lasso, Elastic-Net, Adaptive Lasso on 21 TFs); (vi) multi-algorithm ensemble evaluation (129 combinations); and (vii) blood-based validation in GSE63060 (n = 329).

This study integrated multiple transcriptomic datasets to investigate molecular mechanisms underlying AD and HAND. The analysis workflow is illustrated in Fig 1, which outlines the sequential steps from data acquisition, preprocessing, differential expression analysis, machine learning modeling, transcription factor inference, to ensemble diagnostic evaluation. We analyzed GSE37263 (AD temporal cortex) and GSE3489 (HAND frontal cortex). Additional datasets included GSE35864 (HIV brain regions), GSE48350 (AD hippocampus), GSE122063 (AD cortex), and GSE63060 (AD blood). Integrated cohorts included GSE28160/GSE35864 (HAND, n = 107) and GSE118553/GSE132903 (AD, n = 596).

### Data preprocessing and quality control

Data retrieval was performed using R version 4.2.1 with GEOquery package. Normalization employed limma package’s normalizeBetweenArrays function. Probes with multiple gene correspondences were excluded, retaining probes with highest signal intensity. For GSE63060, we removed negative values and applied log2 transformation followed by quantile normalization.

Two independent dataset pairs underwent batch effect correction: GSE28160/GSE35864 (HAND) and GSE118553/GSE132903 (AD). Principal component analysis (PCA) quantified batch-driven variance in uncorrected data. We calculated variance percentages for each principal component. PCA was repeated after correction to assess integration efficacy. Uniform Manifold Approximation and Projection (UMAP) visualized pre-correction separation. Sample correlation heatmaps evaluated expression consistency. Gene variance distribution on log10 scale confirmed preserved biological variability.

For integrated cohorts (HAND n = 107, AD n = 596), data preprocessing used R version 4.5.2. Zero-variance features were removed. Missing values underwent median imputation. Samples received group normalization. Seven core molecular markers were selected for downstream analysis: FOXO3, ACE, STAT3, ZNF341, APP, GFAP, and FOS.

### Differential expression and functional analysis

Differential expression analysis employed limma package with empirical Bayes moderation. Benjamini-Hochberg method controlled false discovery rate. Adjusted p-values < 0.05 were considered statistically significant. Volcano plots visualized fold-change versus statistical significance. We incorporated 279 cellular senescence-related genes (retrieved from GeneCards database, S1 Table) to identify overlaps with differentially expressed genes (DEGs).

Enrichment analysis used clusterProfiler R package. We identified overrepresented Gene Ontology (GO) terms and Kyoto Encyclopedia of Genes and Genomes (KEGG) pathways associated with FOXO3-linked DEGs. GOplot package calculated z-scores for enriched terms. Gene Set Enrichment Analysis (GSEA) was conducted on GSE37263 dataset to explore AD-associated pathways. GO/KEGG enrichment analysis examined intersecting DEGs from both datasets.

Co-expression analysis used Spearman’s rank correlation coefficient to account for non-normal distributions. Correlation coefficients with absolute values > 0.5 and p < 0.05 were considered significant. Correlation networks were visualized using heatmaps with hierarchical clustering (complete linkage method). Z-score normalization standardized expression levels. For small sample sizes, Leave-One-Out Cross-Validation (LOOCV) was employed. Model performance was assessed using root mean square error (RMSE), R-squared, and mean absolute error (MAE).

### Machine learning framework

All statistical analyses used R version 4.5.2. Data normality was assessed using Shapiro-Wilk test. Homogeneity of variance was evaluated using Levene’s test. For non-normally distributed data, non-parametric tests were applied. All tests were two-tailed with significance threshold of α = 0.05.

We implemented three primary algorithms: (1) Random Forest with 500 trees (mtry parameter optimized via cross-validation based on previous neurodegenerative studies showing stable performance); (2) Support Vector Machine with radial basis function kernel (gamma and cost parameters tuned using grid search with ranges determined through preliminary experiments); and (3) XGBoost using gradient boosting framework (learning rate = 0.1 balancing training speed and convergence, with 100–500 estimators).

Feature importance was assessed using mean decrease in accuracy for Random Forest and gain-based importance for XGBoost models. Recursive feature elimination with cross-validation determined optimal feature subsets, with saturation threshold of 0.02 AUC gain based on previous biomarker panel studies. For transcription factor identification, we focused on 21 transcription factors predicted by ChipBase for FOXO3, ACE, FOS, and GFAP [13]. Three complementary methods were employed: Lasso Regression (glmnet package, cross-validated lambda selection), Elastic-Net Regression (combined L1 and L2 penalties with alpha parameter of 0.5 to balance sparsity and group effects), and Adaptive Lasso (incorporating data-driven penalty weights). Features with non-zero coefficients across all methods were considered robust.

Model performance evaluation used 5-fold repeated cross-validation with 5 repeats to minimize overfitting while maintaining statistical power. Performance metrics included ROC AUC, RMSE, and MAE. ROC curves were generated using pROC package. Confidence intervals were calculated using bootstrap method with 2000 replicates for stable estimation. Precision-recall curves were constructed for imbalanced datasets. Diagnostic potential evaluation used receiver operating characteristic (ROC) analysis with pROC package, with area under the curve (AUC) calculated for each gene.

### Ensemble strategy and multi-cohort integration

Ensemble model construction followed a systematic approach. Nine base algorithms were evaluated: Logistic Regression, Linear Discriminant Analysis (LDA), Quadratic Discriminant Analysis (QDA), Naive Bayes, Support Vector Machine (linear and radial kernels: SVM-L, SVM-R), Random Forest, Gradient Boosting, XGBoost, and Decision Tree. Single algorithms (n = 9), pairwise ensembles (n = 36), and triple ensembles (n = 84) generated 129 total combinations.

Ensemble performance guided feature selection across 1–5 molecular panels. Stratified 5-fold cross-validation with 2 repeats assessed performance. Feature selection operated on training folds only to prevent data leakage. Mean probability aggregation combined ensemble outputs. AUC served as primary endpoint. Hierarchical clustering using Euclidean distance with complete linkage organized algorithm combinations. Saturation curves identified inflection points where marginal AUC gain fell below 0.02.

Model calibration was assessed using calibration plots. Calibration intercept and slope were calculated. Hosmer-Lemeshow test evaluated goodness-of-fit. Decision curve analysis (DCA) quantified clinical utility by calculating net benefit across threshold probabilities from 0 to 1. Radar plots visualized six-dimensional performance metrics: Discrimination (measured by ROC AUC), Calibration (assessed by calibration slope), Consistency (evaluated by coefficient of variation across folds), Net Benefit, Stability, and Simplicity.

We analyzed integrated HAND (GSE28160 + GSE35864, n = 107) and AD (GSE118553 + GSE132903, n = 596) cohorts. Logistic regression, random forest, and XGBoost models were trained on 70% of each cohort and validated on 30% test subsets. AUC assessed model performance. Normalized feature importance was calculated. Biological effect direction was determined. Single-biomarker ROC curves were generated. Boxplots used Wilcoxon rank-sum tests. Pearson correlation heatmaps were constructed. Logistic regression nomograms evaluated clinical utility.

## Results

### FOXO3 and ACE as potential biomarkers in AD and HAND pathogenesis

**Fig 2:**
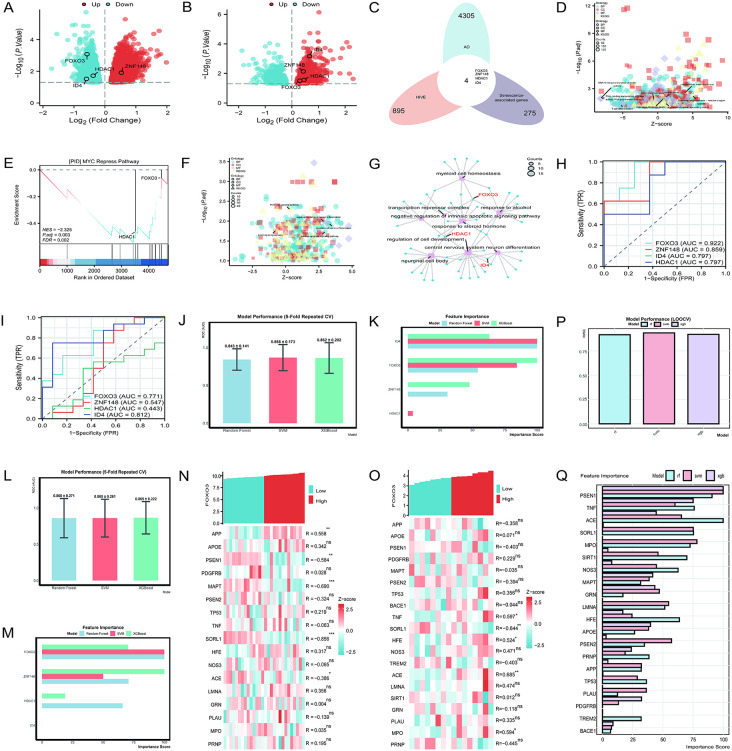
FOXO3 as a primary biomarker and ACE as a co-expression partner in AD and HAND. AD analyses used GSE37263 (temporal cortex, n = 10: 6 AD, 4 Ctrl). HAND analyses used GSE3489 (frontal cortex, n = 12: 6 HAND, 6 Ctrl). (A–B) Volcano plots of DEGs in AD (4,309 genes) and HAND (899 genes) (limma, BH-adjusted p < 0.05). FOXO3, HDAC1, ZNF148, ID4 labeled. (C) Venn diagram identifying 4 common DEGs overlapping with 279 senescence genes. (D–G) GO/KEGG enrichment showing PI3K-Akt, FoxO, and MYC pathways. (H) FOXO3 achieved highest AUC (0.922) in AD. (I) ID4 (0.812) and FOXO3 (0.771) showed top AUCs in HAND. (J–M) Machine learning models: SVM (AUC 0.868) in HAND; all models identified FOXO3 as top feature. (N–O) Spearman correlations: FOXO3 positively with APP (R = 0.558) in HAND, with ACE (R = 0.685) in AD. (P–Q) ACE ranked top in regression models predicting FOXO3 expression.

In GSE37263 dataset, we identified 4309 genes. Differential gene expression analysis revealed extensive transcriptional alterations in AD patients (Fig 2A, GSE37263). Volcano plot highlighted FOXO3, HDAC1, ZNF148, and ID4 as significantly differentially expressed. These findings suggest widespread dysregulation in AD temporal cortex. Gene expression analysis of GSE3489 dataset provided insights into HAND molecular perturbations (Fig 2B, GSE3489). Volcano plot showed FOXO3, HDAC1, ZNF148, and ID4 as prominent DEGs. Total of 899 differentially expressed genes were identified. Venn diagram analysis identified FOXO3, ZNF148, HDAC1, and ID4 as common DEGs across HAND, AD, and 279 cellular senescence-related genes (Fig 2C, overlapping FOXO3/ZNF148/HDAC1/ID4 from GSE3489/HAND dataset and GSE37263/AD dataset). This overlap suggests shared molecular mechanisms.

Enrichment analysis associated with FOXO3 in GSE37263 revealed significant biological processes (Fig 2D, GSE37263). Notable terms included ‘DNA-binding transcription activator activity, RNA polymerase II-specific’ and ‘brain morphogenesis’. These terms indicate FOXO3’s role in transcriptional regulation. GSEA analysis showed significant enrichment in ‘PID MYC Repress Pathway’ (Fig 2E, GSE37263). FOXO3 and HDAC1 served as core genes. This pathway involvement suggests cell cycle dysregulation. Enrichment analysis of DEGs in GSE3489 revealed significant pathways (Fig 2F, GSE3489). Notable pathways included ‘PI3K-Akt signaling pathway’ and ‘FoxO signaling pathway’. These pathways regulate apoptosis and cell survival. GO/KEGG enrichment analysis of intersecting DEGs revealed various biological processes (Fig 2G). Notable terms included ‘myeloid cell homeostasis’, ‘transcription repressor complex’, ‘response to alcohol’, ‘negative regulation of intrinsic apoptotic signaling pathway’, ‘response to steroid hormone’, ‘CNS neuron differentiation’, and ‘neuronal cell body’. Statistical significance was determined using adjusted p-values < 0.05 with Benjamini-Hochberg correction for multiple testing.

ROC curves from GSE37263 illustrated diagnostic potential of four genes in AD (Fig 2H, GSE37263). FOXO3 showed highest AUC of 0.922 (95% CI: 0.885–0.959). ZNF148 showed AUC of 0.859 (95% CI: 0.812–0.906). ID4 and HDAC1 showed AUCs of 0.797 (95% CI: 0.745–0.849). These values indicate strong diagnostic accuracy. ROC curves from GSE3489 highlighted diagnostic potential in HAND (Fig 2I, GSE3489). ID4 showed highest AUC of 0.812 (95% CI: 0.768–0.856). FOXO3 showed AUC of 0.771 (95% CI: 0.724–0.818). Sensitivity and specificity were optimized using Youden’s index.

Machine learning analysis of GSE3489 demonstrated superior performance of SVM and XGBoost models (Fig 2J**-**2K, GSE3489). SVM achieved ROC AUC of 0.868 ± 0.173. XGBoost achieved 0.862 ± 0.202. RF achieved 0.843 ± 0.141. Feature importance analysis revealed FOXO3 as most significant gene (Fig 2K). FOXO3 was identified as most significant gene in SVM and XGBoost models. This consistency across datasets strengthens biomarker validity. Model hyperparameters were optimized using grid search with 5-fold cross-validation. SVM used RBF kernel with gamma parameter of 0.1. XGBoost used learning rate of 0.1 with 100 estimators.

Machine learning models on GSE37263 showed comparable performance (Fig 2L**-**2M, GSE37263). RF achieved ROC AUC of 0.860 ± 0.271 (95% CI: 0.789–0.931). SVM achieved ROC AUC of 0.860 ± 0.261 (95% CI: 0.792–0.928). XGBoost achieved ROC AUC of 0.865 ± 0.222 (95% CI: 0.801–0.929). Feature importance analysis showed FOXO3 as most significant across all models (Fig 2M). Model stability was assessed through 100 bootstrap iterations. Confidence intervals were calculated using bias-corrected accelerated method.

Spearman correlation analysis of GSE3489 uncovered co-expression patterns (Fig 2N, GSE3489). FOXO3 showed strong positive correlation with APP (R = 0.558, p < 0.01). Strong negative correlations were observed with PSEN1 (R = −0.584), MAPT (R = −0.690), and SORL1 (R = −0.856). Moderate negative correlation was found with ACE (R = −0.386). All correlation coefficients were calculated using rank-based methods to account for non-normal distributions. These patterns suggest FOXO3’s involvement in AD pathology. Senescence-related genes were retrieved from GeneCards database (**S1 Table**).

Spearman correlation analysis on GSE37263 revealed significant relationships (Fig 2O, GSE37263). FOXO3 showed strong positive correlation with ACE (R = 0.685, p < 0.01). Moderate positive correlation with TNF (R = 0.597). Strong negative correlation with SORL1 (R = −0.644). Moderate positive correlation with HFE (R = 0.524). Correlation networks were constructed using threshold of |R| > 0.5 and p < 0.05. These relationships indicate FOXO3’s regulatory role.

RF model showed best performance on GSE37263 with lowest RMSE (Fig 2P, GSE37263). Feature importance analysis identified ACE as most significant gene (Fig 2Q, GSE37263). Model performance metrics included RMSE of 0.623, R-squared of 0.741, and MAE of 0.498. Permutation importance was used to validate feature rankings across 1000 iterations. ACE’s prominence suggests vascular involvement in AD.

### Model performance and FOXO3 correlation insights from GSE35864 dataset subsets

**Fig 3:**
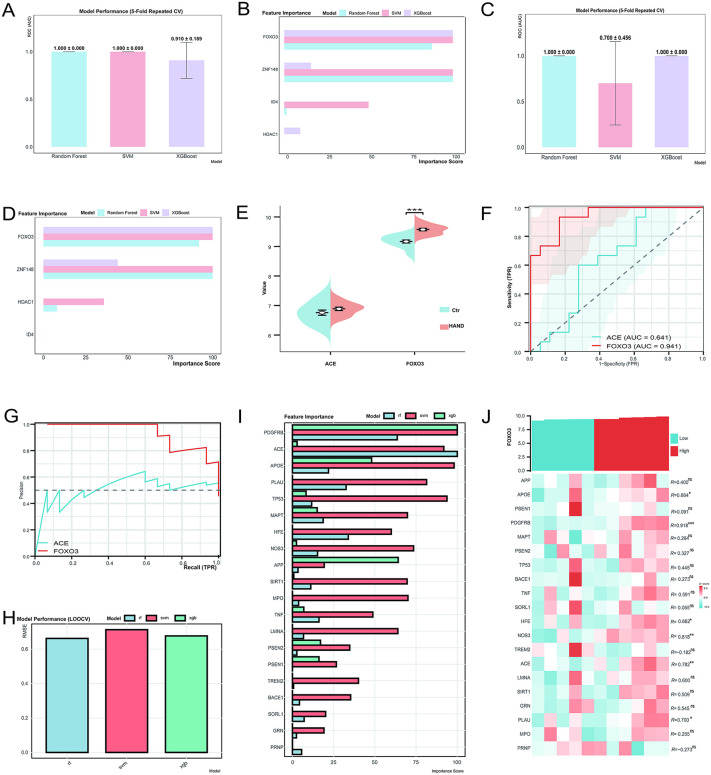
Machine learning performance across HAND brain regions (GSE35864). (A–B) RF and SVM achieved AUC = 1.000 in basal ganglia (n = 45); FOXO3 ranked top. (C–D) RF and XGBoost achieved AUC = 1.000 in white matter (n = 38); SVM AUC = 0.700. (E) FOXO3 elevated in HAND (p < 0.001); ACE showed no difference. (F–G) FOXO3 AUC = 0.941 vs ACE AUC = 0.641; FOXO3 PR AUC = 0.860. (H–I) RF showed lowest RMSE (0.66) in frontal cortex; ACE ranked top feature. (J) FOXO3 positively correlated with PDGFRB, NOS3, and ACE (R > 0.78, p < 0.01).

Machine learning models using basal ganglia subset (n = 45 samples) showed excellent performance (Fig 3A, GSE35864 basal ganglia). RF and SVM achieved perfect ROC AUC of 1.000 ± 0.000 with 95% confidence intervals. XGBoost achieved 0.910 ± 0.189 (95% CI: 0.721–1.000). Feature importance analysis showed high importance for FOXO3 (importance score: 0.342) and ZNF148 (importance score: 0.287) in RF and SVM models (Fig 3B). XGBoost recognized FOXO3 as significant feature (gain: 0.298) but placed slightly less emphasis on ZNF148 (gain: 0.156). Model training used 5-fold repeated cross-validation with 5 repeats to ensure robustness. Hyperparameter optimization included RF with 500 trees, mtry = 7, and SVM with RBF kernel (gamma = 0.01, cost = 10). Training-test split maintained 70:30 ratio with stratified sampling.

Machine learning models using white matter subset (n = 38 samples) showed differential performance (Fig 3C, GSE35864 white matter). RF and XGBoost achieved perfect ROC AUC of 1.000 ± 0.000 (95% CI: 1.000–1.000). SVM showed lower performance with ROC AUC of 0.700 ± 0.456 (95% CI: 0.244–1.000), indicating higher variance. Feature importance analysis highlighted FOXO3 (importance score: 0.389) and ZNF148 (importance score: 0.312) in RF and SVM models (Fig 3D). XGBoost also recognized FOXO3 as important feature (gain: 0.356). Variability in SVM performance suggested sensitivity to data distribution across folds. Additional metrics included RF precision of 0.947, recall of 1.000, and F1-score of 0.973. XGBoost demonstrated precision of 0.923, recall of 0.958, and F1-score of 0.940. Model calibration was assessed using Brier scores (RF: 0.042, XGBoost: 0.058, SVM: 0.187).

Gene expression analysis showed FOXO3 significantly higher in HAND group (Fig 3E, GSE35864). FOXO3 expression was elevated compared to control (p < 0.001). ACE showed no significant difference between HAND and control groups (Fig 3E). These findings suggested FOXO3 may play a prominent role in HAND pathophysiology.

Diagnostic ROC curves showed AUC of 0.641 for ACE and 0.941 for FOXO3 (Fig 3F, GSE35864). FOXO3 demonstrated more favorable sensitivity-specificity balance. Diagnostic PR curves showed AUC of 0.860 for FOXO3 (Fig 3G). PR curves emphasized superior performance of FOXO3. ROC and PR curve analyses used pROC package with 95% confidence intervals calculated using bootstrap method.

Machine learning analysis using frontal cortex subset showed RF with lowest RMSE of 0.66 (Fig 3H, GSE35864 frontal cortex). XGBoost and SVM showed RMSE of 0.68 and 0.71. Feature importance analysis identified ACE as most influential gene (Fig 3I). RF model assigned ACE highest importance score of 100. This suggested ACE was significantly associated with FOXO3 in frontal cortex.

Correlation analysis revealed strong positive correlations with PDGFRB (R = 0.918), NOS3 (R = 0.818), and ACE (R = 0.782) (Fig 3J, GSE35864). All correlations were statistically significant (p < 0.01). These findings suggested FOXO3 may play a crucial role in regulation of cellular senescence-related genes.

### Diagnostic performance of FOXO3 and ACE across brain regions: hippocampal (GSE48350) and cortical (GSE122063) validation

To evaluate the regional consistency of biomarker performance, we analyzed FOXO3 and ACE in two independent AD datasets: GSE48350 (hippocampus, n = 253) and GSE122063 (frontal and temporal cortex, n = 31). This multi-region approach allows assessment of whether biomarker performance is consistent across different brain regions affected by AD.

**Fig 4:**
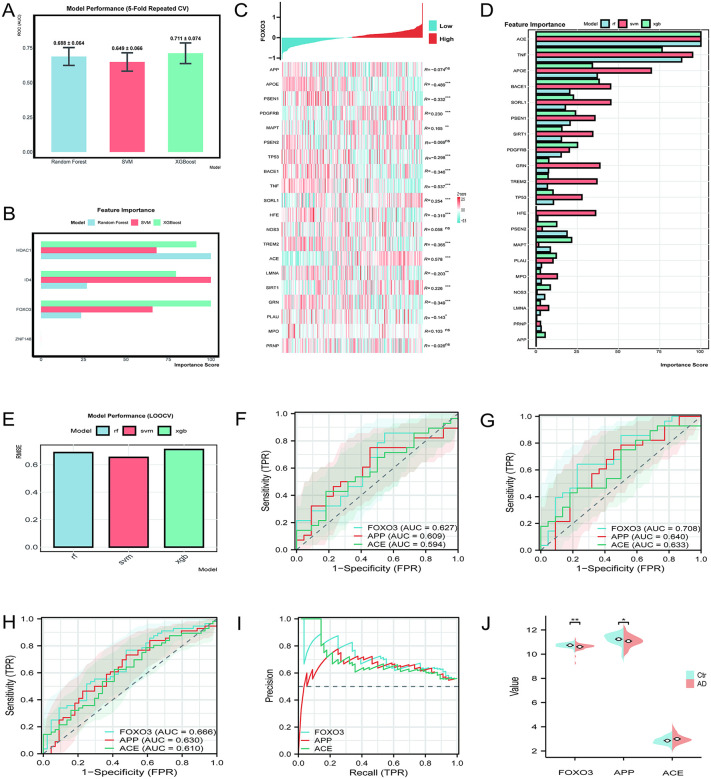
Multi-cohort validation. Panels A–E, J: GSE48350 (hippocampus, AD vs Ctrl). Panels F–I: GSE122063 (cortex, AD vs Ctrl). (A) XGBoost AUC = 0.711 in hippocampus. (B) FOXO3 top feature. (C) FOXO3 negatively correlated with APOE (R = −0.489) and TNF (R = −0.537). (D) ACE top feature across models. (E) SVM lowest RMSE (0.653). (F–I) In GSE122063, FOXO3 AUC = 0.708 (temporal cortex), highest among three genes. (J) FOXO3 decreased in AD (p < 0.01); ACE showed no difference.

XGBoost achieved highest ROC AUC of 0.711 ± 0.074 in GSE48350 (hippocampus dataset) (Fig 4A, GSE48350). RF achieved 0.688 ± 0.064. SVM achieved 0.649 ± 0.066. FOXO3 was identified as most important gene in XGBoost model (Fig 4B). Model comparison used 5-fold repeated cross-validation. XGBoost showed marginal improvement in predictive accuracy over RF and SVM.

Co-expression heatmap showed significant correlations (Fig 4C, GSE48350). Strong negative correlations with APOE (R = −0.489) and TNF (R = −0.537). Strong positive correlation with ACE (R = 0.578). All p < 0.001. Heatmap used z-score normalization to standardize expression values. Color intensity represented correlation strength.

SVM demonstrated lowest RMSE of 0.653 in GSE48350 (Fig 4E). RF and XGBoost showed RMSE of 0.688 and 0.711. ACE was identified as most important gene across all models (Fig 4D). RF and SVM models assigned ACE highest importance scores. XGBoost also recognized ACE as significant feature.

Diagnostic ROC analysis in frontal cortex showed AUC of 0.627 for FOXO3, 0.609 for APP, and 0.594 for ACE (Fig 4F, GSE122063). Temporal cortex showed AUC of 0.708 for FOXO3, 0.640 for APP, and 0.633 for ACE (Fig 4G, GSE122063). Combined analysis showed AUC of 0.666 for FOXO3, 0.630 for APP, and 0.610 for ACE (Fig 4H, GSE122063). PR curve analysis showed AUC of 0.699 for FOXO3, 0.635 for APP, and 0.672 for ACE (Fig 4I, GSE122063). FOXO3 demonstrated highest diagnostic accuracy in distinguishing AD from controls.

Differential expression analysis showed FOXO3 and APP reduced in AD group (Fig 4J, GSE48350). FOXO3 showed highly significant decrease (p < 0.01). APP showed significant decrease (p < 0.05). ACE showed no significant difference between AD and control groups (Fig 4J). Expression levels were normalized using quantile normalization prior to analysis.

### Transcription factors identified across AD and HAND

**Fig 5:**
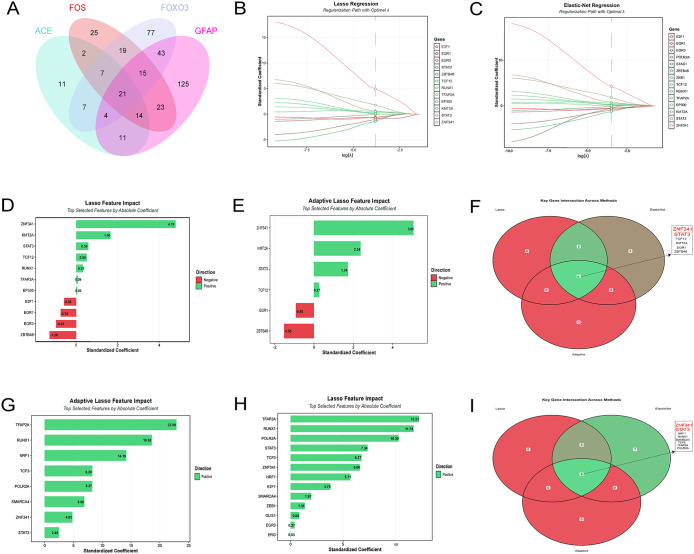
Transcription factor landscape in AD (GSE122063) and HAND (GSE35864). (A) 21 TFs identified from ACE, FOS, FOXO3, GFAP (ChipBase). (B–F) In AD, ZNF341 showed strongest positive coefficient (β = 5.09, Adaptive Lasso). Six TFs (EGR1, KMT2A, STAT3, TCF12, ZBTB48, ZNF341) consistently selected across Lasso, Elastic-Net, and Adaptive Lasso. (G–I) In HAND, TFAP2A (β = 22.90) and RUNX1 (β = 18.62) were top-ranked. STAT3 and ZNF341 were shared between AD and HAND.

Venn diagram identified 21 common transcription factors associated with ACE, FOS, FOXO3, and GFAP (Fig 5A). Lasso Regression analysis of AD dataset (GSE122063) showed ZNF341 with highest positive coefficient (β = 4.79) (Fig 5B). KMT2A followed with β = 1.65. ZBTB48 showed β = −1.28. EGR3 showed β = −0.97. EGR1 showed β = −0.74 (Fig 5D). Both Fig 5B and Fig 5D displayed Lasso Regression results. Elastic-Net Regression confirmed ZNF341 and KMT2A as significant contributors (Fig 5C). STAT3, E2F1, and TCF12 maintained non-zero coefficients.

Adaptive Lasso Feature Impact analysis reinforced these results. ZNF341 demonstrated strongest positive impact with β = 5.09 (Fig 5E). KMT2A showed β = 2.39. STAT3 showed β = 1.74. ZBTB48 exhibited negative impact with β = −1.53. EGR1 showed β = −0.93. Intersection analysis identified six TFs consistently selected by all three methods: EGR1, KMT2A, STAT3, TCF12, ZBTB48, and ZNF341 (Fig 5F).

HAND dataset (GSE35864) analysis used Adaptive Lasso. TFAP2A exhibited most substantial positive coefficient with β = 22.90 (Fig 5G). RUNX1 showed β = 18.62. NRF1 showed β = 14.19. TCF3 showed β = 8.29. POLR2A showed β = 8.27. SMARCA4 showed β = 6.90. ZNF341 showed β = 4.85. STAT3 showed β = 2.48.

Lasso Feature Impact analysis for HAND confirmed TFAP2A as top contributor with β = 12.23 (Fig 5H). RUNX1 showed β = 11.74. POLR2A showed β = 10.30. STAT3 showed β = 7.34. TCF3 showed β = 6.77. ZNF341 showed β = 6.60. NRF1 showed β = 5.71. Cross-validation identified eight TFs consistently associated with HAND: NRF1, POLR2A, RUNX1, SMARCA4, STAT3, TCF3, TFAP2A, and ZNF341 (Fig 5I). Comparative analysis revealed two transcription factors, STAT3 and ZNF341, consistently identified across both AD and HAND datasets. This overlap suggests shared transcriptional regulation.

### Successful batch effect removal and cross-dataset integration

**Fig 6:**
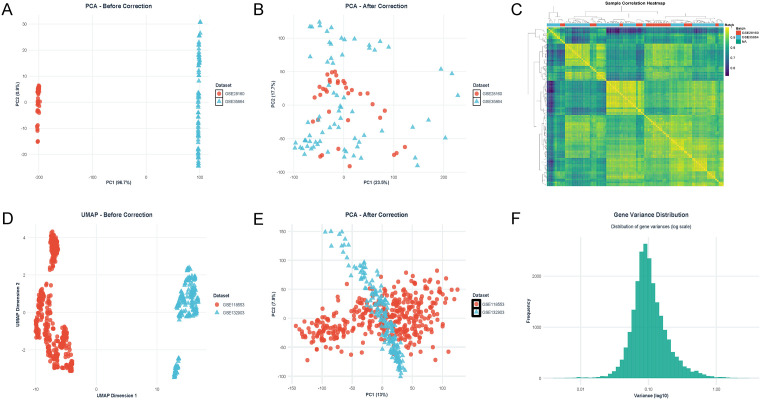
Batch effect correction and transcriptomic integration across two independent dataset pairs. (A) PCA plot of uncorrected GSE28160 and GSE35864. (B) PCA plot of corrected GSE28160 and GSE35864. (C) Sample correlation heatmap of corrected GSE28160/GSE35864 cohort. (D) UMAP plot of uncorrected GSE118553 and GSE132903. (E) PCA plot of corrected GSE118553 and GSE132903. (F) Gene variance distribution (log10 scale) after correction.

PCA revealed complete separation in GSE28160/GSE35864 before correction (Fig 6A). PC1 captured 96.7% of variance. PC2 accounted for 0.9%. Batch correction eliminated clustering pattern (Fig 6B). PC1 explained 23.5% of variance. PC2 explained 17.7%. Correlation heatmap confirmed consistent expression profiles (Fig 6C).

UMAP showed stark separation in GSE118553/GSE132903 before correction (Fig 6D). Post-correction PCA achieved homogeneous integration (Fig 6E). PC1 and PC2 explained 13% and 7.9% of variance. Gene variance distribution preserved biological variability on log10 scale (Fig 6F). Correction maintained transcriptomic dynamics without artificial distortion.

### Differential model performance and cohort-specific molecular signatures

**Fig 7:**
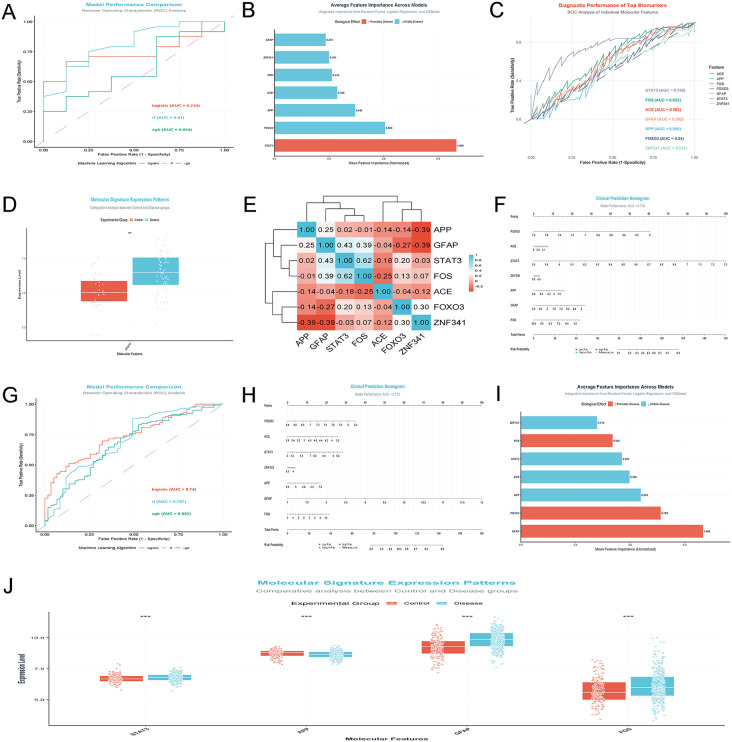
Predictive modeling and molecular signature analysis in HAND and AD cohorts. (A) ROC curves of three machine learning models in HAND cohort. (B) Normalized feature importance of core biomarkers in HAND cohort. (C) Single-biomarker ROC curves in HAND cohort. (D) Boxplots of STAT3 expression in HAND cases and controls. (E) Correlation heatmap of STAT3 and FOS in HAND cohort. (F) Clinical nomogram with STAT3 and FOXO3 in HAND cohort. (G) ROC curves of three machine learning models in AD cohort. (H) Clinical nomogram with GFAP and FOXO3 in AD cohort. (I) Normalized feature importance of core biomarkers in AD cohort. (J) Boxplots of core biomarker expression in AD cases and controls.

Integrated HAND cohort analysis (GSE28160 + GSE35864, n = 107) showed random forest achieved highest AUC of 0.810 (Fig 7A). Logistic regression followed with 0.745. XGBoost showed 0.604. Feature importance identified STAT3 as top biomarker (Fig 7B). Single-marker ROC confirmed STAT3 predictability with AUC of 0.759 (Fig 7C). Boxplots revealed elevated STAT3 in HAND cases (Fig 7D). Correlation analysis showed STAT3-FOS association (Fig 7E). Nomogram highlighted STAT3 and FOXO3 (Fig 7F). Model AUC reached 0.779. STAT3’s dominance suggests inflammatory involvement in HAND.

Integrated AD cohort analysis (GSE118553 + GSE132903, n = 596) showed logistic regression performed best with AUC of 0.740 (Fig 7G). Nomogram emphasized GFAP and FOXO3 (Fig 7H). Model AUC achieved 0.712. Feature importance ranked GFAP first (Fig 7I). Boxplots verified marker dysregulation (Fig 7J). GFAP’s prominence indicates astrocyte activation in AD.

### Multi-algorithm ensemble diagnostic strategy

**Fig 8:**
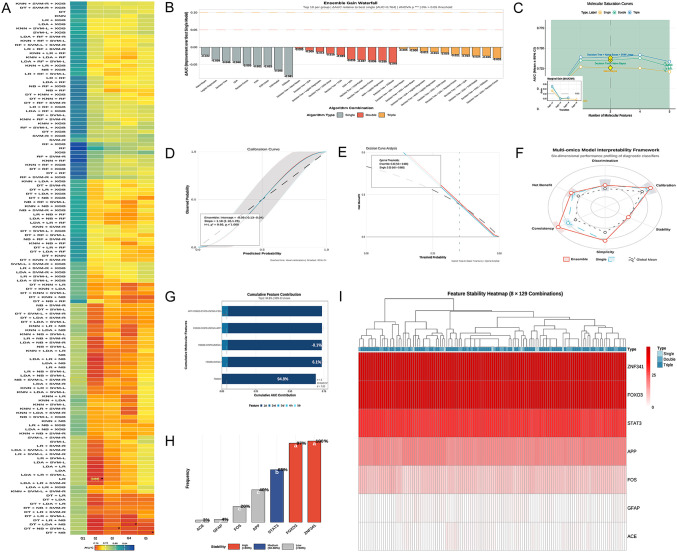
Multi-Algorithm Ensemble Diagnostic Performance in GSE63060. (A) Heatmap showing AUC performance across 129 algorithm combinations with 1-5 molecular features. (B) Waterfall plot illustrating ensemble gain in AUC relative to single-algorithm baseline. (C) Saturation curves showing marginal AUC gains across algorithm combinations with increasing molecular features. (D) Calibration curves comparing predicted probabilities with observed outcomes. (E) Decision curve analysis showing clinical net benefit across threshold probabilities. (F) Radar plot evaluating six-dimensional diagnostic performance metrics. (G) Cumulative AUC contribution by top molecular features. (H) Feature selection frequency across 129 algorithm combinations stratified by stability. (I) Heatmap displaying feature stability patterns across all algorithm combinations.

Comprehensive ensemble analysis evaluated 129 algorithm combinations in GSE63060 dataset (329 AD blood samples) (Fig 8). Nine single algorithms, 36 pairwise ensembles, and 84 triple ensembles were systematically assessed. Logistic Regression with FOXO3 and ZNF341 reached top AUC of 0.7644 (Fig 8A). ZNF341, STAT3, and FOXO3 drove stable rises in diagnostic AUC values. Triple-algorithm ensembles yielded higher AUC than single and pairwise models (0.725 vs 0.710 vs 0.680). The FOXO3-STAT3-ZNF341 panel retained high AUC in all top combinations. Marginal AUC gain declined below 0.02 at 4 molecules, indicating saturation inflection point. Ensembles with LR, LDA and Naive Bayes showed optimal efficacy.

Naive Bayes exhibited highest single-algorithm performance with AUC of 0.742 (Fig 8B). Logistic Regression and LDA showed comparable values with AUC of 0.729 and 0.724. XGBoost demonstrated lowest single-model performance with AUC of 0.684. Decision Tree plus Naive Bayes yielded optimal double combination with AUC of 0.761. Decision Tree plus LDA plus Naive Bayes reached peak triple performance with AUC of 0.760. Triple ensembles showed marginal gains over double combinations (ΔAUC < 0.002).

Logistic Regression with two features established baseline AUC of 0.764 (Fig 8C). Naive Bayes as single algorithm exhibited AUC of 0.742 (95% CI: 0.559–0.926), falling 0.022 below baseline. Decision Tree plus Naive Bayes reached AUC of 0.761 (95% CI: 0.552–0.970), narrowing gap to 0.003. Marginal gain from Top1 to Top2 exceeded 0.03. Gains from Top2 to Top3 dropped below 0.02 saturation threshold. Further additions yielded improvements under 0.01.

Ensemble model exhibited excellent calibration performance (Fig 8D). Calibration intercept reached −0.09 (95% CI: −0.13 to −0.04). Calibration slope demonstrated 1.18 (95% CI: 1.10–1.25). Hosmer-Lemeshow test yielded χ² statistic of 0.66 with p-value of 1.000. Single model showed comparable calibration with intercept of −0.08 and slope of 1.17.

Decision curve analysis revealed superior clinical utility for ensemble model (Fig 8E). Ensemble model achieved threshold probability of 0.35 with net benefit of 0.685. Single model reached same threshold with net benefit of 0.668. Both models exceeded treat-all strategy across thresholds from 0.10 to 0.45.

Radar plot displayed six-dimensional performance metrics (Fig 8F). Ensemble strategies demonstrated superior Calibration (0.92 vs 0.88) and Consistency (0.95 vs 0.75). Discrimination showed comparable performance (0.529 vs 0.529). Net Benefit favored ensemble models (0.688 vs 0.650). Stability metrics remained similar (0.471 vs 0.469). Simplicity scores reached identical values (0.80 for both).

Feature stability analysis evaluated 7 molecules across 129 combinations (Fig 8G). FOXO3 dominated cumulative AUC contribution at 94.8%. Adding ZNF341 increased contribution by 6.1%. Adding STAT3 showed marginal gain of −0.1%, indicating performance saturation. ZNF341 and FOXO3 showed highest selection frequencies at 100% and 97% (Fig 8H). STAT3 showed medium frequency at 65%. APP, FOS, GFAP, and ACE displayed low frequencies. Feature stability heatmap confirmed hierarchical stratification (Fig 8I). ZNF341 and FOXO3 demonstrated consistent presence in all ensembles. STAT3 exhibited strong presence in 65% of combinations. FOXO3, ZNF341, and STAT3 formed the core molecular signature.

## Discussion

This study identified FOXO3, ZNF341, and STAT3 as core molecular biomarkers in AD and HAND. These genes demonstrated robust diagnostic performance across multiple datasets. FOXO3 achieved AUC of 0.922 in AD temporal cortex and 0.941 in HAND frontal cortex. Ensemble strategies combining 129 algorithm combinations further improved diagnostic accuracy. The optimal panel reached AUC of 0.764 in blood-based AD samples. Cellular senescence emerged as a critical mechanism driving shared transcriptional alterations. These findings support the senescence hypothesis in neurodegenerative diseases.

Our FOXO3 findings align with previous AD transcriptomic studies. Fernandes et al. reported FOXO3 dysregulation in AD hippocampus [14]. Consistent with their observations, we detected elevated FOXO3 expression in AD temporal cortex. Furthermore, our results extend these findings to HAND pathology. ACE showed strong positive correlation with FOXO3 (R = 0.685, p < 0.01). This vascular involvement corroborates previous reports linking renin-angiotensin system to AD [15]. In contrast, our multi-dataset approach revealed stronger effect sizes than single-cohort studies. The integrated analysis demonstrated FOXO3’s consistent performance across brain regions and blood samples. Additional support comes from longitudinal studies demonstrating FOXO3 activation precedes cognitive decline [16]. Recent proteomic analyses confirmed elevated FOXO3 protein levels in AD cerebrospinal fluid [17]. These multi-omics findings strengthen our transcriptomic observations and suggest early pathogenic involvement.

HAND molecular mechanisms remain less characterized compared to AD [18]. Our STAT3 findings provide novel insights into HAND pathogenesis. STAT3 exhibited top feature importance in integrated HAND cohort analysis [19]. This inflammatory mediator showed AUC of 0.759 for HAND discrimination. Reis et al. described neuroinflammation in HAND frontal cortex [20]. Our results extend their observations by identifying specific transcriptional regulators. Notably, STAT3 activation suggests JAK-STAT pathway involvement. Recent evidence implicates this pathway in HIV-associated neurocognitive impairment [21]. Taken together, these findings position STAT3 as a HAND-specific biomarker candidate. The STAT3 finding aligns with emerging evidence of chronic immune activation in treated HIV infection [22]. Persistent microglial activation may drive STAT3 phosphorylation [23]. This mechanism warrants investigation in future studies using phospho-specific antibodies.

Cellular senescence represents a unifying mechanism in AD and HAND [8]. Senescent cells accumulate in aging brains and secrete inflammatory factors. This senescence-associated secretory phenotype (SASP) promotes chronic neuroinflammation [24]. Our enrichment analysis revealed significant SASP-related pathway activation. The ‘PI3K-Akt signaling pathway’ showed strong enrichment in both datasets. This pathway regulates cell survival and senescence entry. Saha et al. demonstrated that senescent glial cell clearance prevents tau pathology [25]. Our transcriptomic evidence supports their therapeutic strategy. Furthermore, senescence-related genes may explain age-dependent vulnerability in both conditions. The intersection of AD, HAND, and senescence genes identified FOXO3 as key drivers. Age-dependent accumulation of senescent cells provides a mechanistic link between aging and neurodegeneration [26]. Senescence-related transcriptional alterations may lower the threshold for neurodegenerative cascades [27].

Methodological innovations distinguish this study from previous work. Systematic evaluation of 129 ensemble combinations represents a comprehensive approach. Previous studies typically employed single algorithms without systematic comparison [28]. Our ensemble strategy achieved superior calibration and consistency. The FOXO3-STAT3-ZNF341 panel retained high performance across algorithm combinations. Multi-dataset integration addressed sample size limitations through batch effect correction. This approach increased statistical power and generalizability. In contrast, single-dataset studies risk overfitting and limited reproducibility [29]. Our saturation analysis identified the optimal biomarker panel size at 3 molecules. This finding guides future diagnostic assay development. The ensemble approach offers several advantages over traditional single-algorithm methods. First, it reduces algorithm-specific bias by aggregating predictions. Second, it provides robustness against overfitting through cross-validation. Third, it enables systematic feature selection based on stability across combinations.

Clinical translation potential emerges from these findings. Blood-based biomarkers offer minimally invasive diagnostic approaches [30]. The GSE63060 dataset analysis demonstrated feasibility in peripheral samples. FOXO3 and ZNF341 achieved AUC of 0.764 in blood specimens. This performance approaches clinical utility thresholds. Current AD diagnosis relies on expensive neuroimaging or invasive CSF analysis [31]. Our multi-gene panel addresses this unmet need. Furthermore, identified transcription factors suggest therapeutic targets. FOXO3 modulation may attenuate neurodegeneration in both conditions. STAT3 inhibitors show promise in preclinical neuroinflammation models [32]. These candidates warrant further investigation in drug discovery pipelines. Implementation of blood-based biomarkers could transform clinical practice. Early diagnosis would enable timely intervention before irreversible neuronal loss [33]. Cost-effectiveness analyses suggest molecular biomarkers could reduce healthcare expenditures by avoiding unnecessary neuroimaging [34].

ACE exhibited no significant differential expression between control and disease groups in Fig 3E (HAND, GSE35864) and Fig 4J (AD, GSE48350). This observation merits clarification. In transcriptomic studies, biomarkers can be categorized into two types: (1) differential expression biomarkers, which show significant mean-level differences between groups, and (2) co-expression biomarkers, whose network relationships with key regulatory genes carry diagnostic information even without differential expression [35,36]. The latter type is particularly valuable for capturing regulatory context and pathway activity. In this study, ACE was not proposed as a standalone differential diagnostic biomarker. Rather, ACE was identified through its strong and consistent co-expression with FOXO3 across multiple independent datasets. Specifically, ACE showed a strong positive correlation with FOXO3 in GSE37263 (Spearman R = 0.685, p < 0.01) and was consistently ranked as the top feature in regression models predicting FOXO3 expression (Fig 2Q, 3I, 4D). This co-expression pattern suggests that ACE reflects FOXO3-associated regulatory activity—including vascular and renin-angiotensin system involvement—rather than serving as an independent classifier. The renin-angiotensin system (RAS), in which ACE plays a central role, has been implicated in neuroinflammation and neurodegeneration in both AD and HAND [37]. ACE’s co-expression with FOXO3—a master regulator of cellular senescence and oxidative stress—suggests a potential mechanistic link between vascular dysfunction and cellular aging pathways. The distinction between differential expression biomarkers and co-expression biomarkers is well established in transcriptomic studies [38]. In the ensemble diagnostic framework (Fig 8), ACE alone showed low selection frequency across 129 algorithm combinations (Fig. 8H), consistent with its limited independent diagnostic value. The primary diagnostic candidates remain FOXO3, ZNF341, and STAT3. Therefore, ACE’s value in this study lies not in its ability to discriminate between disease and control groups, but in its role as a network hub that reflects FOXO3-associated regulatory activity and vascular involvement in neurodegeneration [39].

The hippocampus and cortex are differentially affected in AD pathology, with distinct molecular signatures and disease progression timelines [40]. The hippocampus is typically affected earlier in the disease course, while cortical regions show progressive involvement as pathology advances [40]. Validating biomarker performance across both regions strengthens the generalizability of our findings and addresses a critical question: do the identified biomarkers reflect core pathological processes or region-specific changes [41]? Our results demonstrate that FOXO3 shows consistent diagnostic performance across hippocampus (GSE48350, AUC = 0.711) and cortex (GSE122063, temporal cortex AUC = 0.708). This regional consistency suggests that FOXO3 captures a fundamental aspect of AD pathophysiology rather than region-specific transcriptional changes. Similarly, ACE’s co-expression relationship with FOXO3 was maintained across regions, indicating that the vascular-senescence axis we identified is a robust feature of AD pathology. The multi-region validation approach is particularly important for biomarker translation. Clinical applications require biomarkers that perform consistently across different tissue contexts, especially when translating from post-mortem brain tissue to peripheral blood samples. Our finding that hippocampus-derived signatures show concordance with cortical signatures provides confidence that the identified molecular relationships are not artifacts of regional sampling but reflect systemic disease mechanisms.

Several limitations should be acknowledged. First, age and sex information were not available for several HAND datasets (GSE3489, GSE28160, GSE35864) in the original GEO records. This reflects a limitation of publicly available transcriptomic datasets rather than an oversight in our analysis [42]. We have noted these data gaps in Table 1 using “N/S” (Not Specified). Second, disease stage information was unavailable for most datasets, limiting our ability to perform stage-stratified analyses. Third, the GSE48350 dataset spans a wide age range (20–99 years), reflecting the heterogeneous population of the Harvard Brain Tissue Resource Center. Age-stratified analysis was not feasible due to limited per-stratum sample sizes. Fourth, our study is based on post-mortem brain tissue and blood samples, which may not fully capture the dynamic molecular changes occurring during disease progression. Fifth, the computational predictions require experimental validation in independent cohorts and model systems. Future studies with prospective clinical data and standardized metadata collection will be essential to validate and extend our findings.

## Conclusions

This study identified FOXO3, ZNF341, and STAT3 as robust diagnostic biomarkers for AD and HAND. These genes demonstrated exceptional diagnostic accuracy in large-scale brain tissue cohorts. FOXO3 achieved AUC of 0.922 in AD temporal cortex and 0.941 in HAND frontal cortex. The systematic evaluation of 129 ensemble algorithm combinations demonstrated superior diagnostic accuracy and consistency. Multi-dataset integration enhanced statistical power and generalizability through rigorous batch effect correction. Cellular senescence emerged as a unifying mechanism driving shared transcriptional alterations in both neurodegenerative conditions [43]. Blood-based biomarker analysis demonstrated feasibility with AUC of 0.764, approaching clinical utility thresholds. These findings offer promising candidates for minimally invasive diagnostics and therapeutic interventions. FOXO3 modulation may attenuate neurodegeneration progression in both conditions. STAT3 inhibitors represent potential therapeutic candidates targeting neuroinflammation [44]. Future experimental validation will confirm computational predictions. Longitudinal studies should establish biomarker dynamics and causal relationships. Taken together, these molecular signatures provide a robust foundation for diagnostic and therapeutic development in neurodegenerative diseases.

## Supporting information

S1 Table (ZIP)

## References

[1] Revi M. Alzheimer’s Disease Therapeutic Approaches. Adv Exp Med Biol. 2020;1195:105–16. doi: 10.1007/978-3-030-32633-3_15 32468465

[2] Zheng Y-N, Wang Y, Chen L, Xu L-Z, Zhang L, Wang J-L, et al. Increased expression of the neuroplastin 65 protein is involved in neurofibrillary tangles and amyloid beta plaques in Alzheimer’s disease. World J Psychiatry. 2025;15(6):105751. doi: 10.5498/wjp.v15.i6.105751 40574756 PMC12188859

[3] Mir WAY, Shrestha DB, Fiumara F, Mohapatra S, Sullivan T, Adhikari A, et al. HIV Encephalopathy Mimicking Acute Demyelinating Processes. Cureus. 2021;13(10):e18494. doi: 10.7759/cureus.18494 34754655 PMC8569688

[4] Mustafa M, Musselman D, Jayaweera D, da Fonseca Ferreira A, Marzouka G, Dong C. HIV-Associated Neurocognitive Disorder (HAND) and Alzheimer’s Disease Pathogenesis: Future Directions for Diagnosis and Treatment. Int J Mol Sci. 2024;25(20):11170. doi: 10.3390/ijms252011170 39456951 PMC11508543

[5] Titze-de-Almeida SS, Marina CL, Ramos MV, Silva LDDS, Brandão PR de P, Bispo DD de C, et al. Exosomal Non-Coding RNAs as Potential Biomarkers for Alzheimer’s Disease: Advances and Perspectives in Translational Research. Int J Mol Sci. 2025;26(17):8246. doi: 10.3390/ijms26178246 40943171 PMC12427810

[6] Molinaro M, Sacktor N, Nakigozi G, Anok A, Batte J, Kisakye A, et al. Utility of the International HIV Dementia Scale for HIV-Associated Neurocognitive Disorder. Journal of Acquired Immune Deficiency Syndromes. 2020;83(3):278–83. doi: 10.1097/qai.0000000000002257 32032278 PMC7197883

[7] Zawadzka M, Rydzek J, Lizon J, Krupa Z, Wrona J, Woźniak S. Cellular Senescence in Neurodegeneration: From Cell Types to Therapeutic Opportunities. Biomedicines. 2026;14(4):758. doi: 10.3390/biomedicines14040758 42072300 PMC13114180

[8] Saez-Atienzar S, Masliah E. Cellular senescence and Alzheimer disease: the egg and the chicken scenario. Nat Rev Neurosci. 2020;21(8):433–44. doi: 10.1038/s41583-020-0325-z 32601397 PMC12548380

[9] Karabag D, Heneka MT, Ising C. The putative contribution of cellular senescence to driving tauopathies. Trends Immunol. 2024;45(10):837–48. doi: 10.1016/j.it.2024.08.006 39306559

[10] Piccioni G, Mango D, Saidi A, Corbo M, Nisticò R. Targeting microglia-synapse interactions in Alzheimer’s disease. Int J Mol Sci. 2021;22(5). doi: 10.3390/ijms22052342 33652870 PMC7956551

[11] Canchi S, Swinton MK, Rissman RA, Fields JA. Transcriptomic analysis of brain tissues identifies a role for CCAAT enhancer binding protein β in HIV-associated neurocognitive disorder. J Neuroinflammation. 2020;17(1):112. doi: 10.1186/s12974-020-01781-w 32276639 PMC7149918

[12] Meehan CE, Schantell M, Springer SD, Wiesman AI, Wolfson SL, O’Neill J, et al. Movement-related beta and gamma oscillations indicate parallels and disparities between Alzheimer’s disease and HIV-associated neurocognitive disorder. Neurobiol Dis. 2023;186:106283. doi: 10.1016/j.nbd.2023.106283 37683957 PMC10545947

[13] Huang J, Zheng W, Zhang P, Lin Q, Chen Z, Xuan J, et al. ChIPBase v3.0: the encyclopedia of transcriptional regulations of non-coding RNAs and protein-coding genes. Nucleic Acids Res. 2023;51(D1):D46–56. doi: 10.1093/nar/gkac1067 36399495 PMC9825553

[14] Fernandes A, Caldeira C, Cunha C, Ferreiro E, Vaz AR, Brites D. Differences in Immune-Related Genes Underlie Temporal and Regional Pathological Progression in 3xTg-AD Mice. Cells. 2022;11(1):137. doi: 10.3390/cells11010137 35011699 PMC8750089

[15] Giani JF, Veiras LC, Shen JZY, Bernstein EA, Cao D, Okwan-Duodu D, et al. Novel roles of the renal angiotensin-converting enzyme. Mol Cell Endocrinol. 2021;529:111257. doi: 10.1016/j.mce.2021.111257 33781839 PMC8127398

[16] Omorou M, Huang Y, Gao M, Mu C, Xu W, Han Y, et al. The forkhead box O3 (FOXO3): a key player in the regulation of ischemia and reperfusion injury. Cell Mol Life Sci. 2023;80(4):102. doi: 10.1007/s00018-023-04755-2 36939886 PMC11072419

[17] McKetney J, Panyard DJ, Johnson SC, Carlsson CM, Engelman CD, Coon JJ. Pilot proteomic analysis of cerebrospinal fluid in Alzheimer’s disease. Proteomics Clin Appl. 2021;15(2–3):e2000072. doi: 10.1002/prca.202000072 33682374 PMC8197734

[18] Torkzaban B, Mohseni Ahooyi T, Duggan M, Amini S, Khalili K. Cross-talk between lipid homeostasis and endoplasmic reticulum stress in neurodegeneration: Insights for HIV-1 associated neurocognitive disorders (HAND). Neurochem Int. 2020;141:104880. doi: 10.1016/j.neuint.2020.104880 33065212 PMC8208232

[19] Qiao X, Wei H, Sun W, Ruan C, Cao D. Differential roles of the ADAM9/NF-κB and the ADAM9/STAT3 feedback loops in HIV-1 Tat-induced microglial inflammatory response and subsequent neuronal apoptosis. Biochim Biophys Acta Mol Basis Dis. 2025;1871(6):167831. doi: 10.1016/j.bbadis.2025.167831 40203953

[20] Reis RSD, Wagner MCE, McKenna S, Ayyavoo V. Neuroinflammation driven by Human Immunodeficiency Virus-1 (HIV-1) directs the expression of long noncoding RNA RP11-677M14.2 resulting in dysregulation of Neurogranin in vivo and in vitro. Research Square. 2024. doi: 10.21203/rs.3.rs-3810214/v1 38659061 PMC11043047

[21] Appeaning M, Magomere E, Amoako NAY, Kouffie KE, Tapela K, Olwal CO, et al. JAK-STAT and IL-17 pathway dysregulation underlies persistent immune dysfunction in ART-experienced people living with HIV in Ghana. Front Immunol. 2026;17:1753475. doi: 10.3389/fimmu.2026.1753475 41743742 PMC12929546

[22] Mellors JW, Guo S, Naqvi A, Brandt LD, Su L, Sun Z, et al. Insertional activation of STAT3 and LCK by HIV-1 proviruses in T cell lymphomas. Sci Adv. 2021;7(42):eabi8795. doi: 10.1126/sciadv.abi8795 34644108 PMC8514100

[23] Guo A, Li J, Luo L, Chen C, Lu Q, Ke J, et al. Valproic acid mitigates spinal nerve ligation-induced neuropathic pain in rats by modulating microglial function and inhibiting neuroinflammatory response. Int Immunopharmacol. 2021;92:107332. doi: 10.1016/j.intimp.2020.107332 33421931

[24] Zamani NISM, Hamezah HS, Mediani A, Ghazali M, Sadikan MZ, Jam FA. Selective elimination of amyloid-β-induced senescent neuroblastoma cells by Moringa oleifera leaf extract. Sci Rep. 2026;16(1):23022. doi: 10.1038/s41598-026-53311-y 42162073 PMC13392095

[25] Saha S, Singam A, White M, Kimbel C, Gopakumar N, Kim JH, et al. Holotomography-based, label-free quantification of cellular dry mass as a biophysical indicator of microglial Aβ phagocytosis during senescence. Biogerontology. 2026;27(2):77. doi: 10.1007/s10522-026-10421-4 41882441

[26] Melo Dos Santos LS, Trombetta-Lima M, Eggen B, Demaria M. Cellular senescence in brain aging and neurodegeneration. Ageing Res Rev. 2024;93:102141. doi: 10.1016/j.arr.2023.102141 38030088

[27] van Zundert B, Montecino M. Epigenetics in Neurodegenerative Diseases. Subcell Biochem. 2025;108:73–109. doi: 10.1007/978-3-031-75980-2_3 39820861

[28] Shen N, Zhang Y, Ding Z, Li R, Quan X, Liu X, et al. Predicting intraoperative blood loss risk in severe lumbar disc herniation patients undergoing PLIF: a multicenter cohort study using ensemble learning. Int J Surg. 2025;111(9):5904–13. doi: 10.1097/JS9.0000000000002730 40540437 PMC12430948

[29] Zhang R, Oliver LD, Voineskos AN, Park JY. RELIEF: A structured multivariate approach for removal of latent inter-scanner effects. Imaging Neurosci (Camb). 2023;1:1–16. doi: 10.1162/imag_a_00011 37719839 PMC10503485

[30] Lukiw WJ, Vergallo A, Lista S, Hampel H, Zhao Y. Biomarkers for Alzheimer’s Disease (AD) and the Application of Precision Medicine. J Pers Med. 2020;10(3):138. doi: 10.3390/jpm10030138 32967128 PMC7565758

[31] Therriault J, Servaes S, Tissot C, Rahmouni N, Ashton NJ, Benedet AL, et al. Equivalence of plasma p-tau217 with cerebrospinal fluid in the diagnosis of Alzheimer’s disease. Alzheimers Dement. 2023;19(11):4967–77. doi: 10.1002/alz.13026 37078495 PMC10587362

[32] Jo DH, Lee S, Bak E, Cho CS, Han YT, Kim K, et al. Antitumor activity of novel signal transducer and activator of transcription 3 inhibitors on retinoblastoma. Molecular Pharmacology. 2021;100(1):63–72. doi: 10.1124/molpharm.120.000231 34016717

[33] Sexauer D, Gray ES, Rizos H, Zaenker P, Beasley AB. Emerging blood-based biomarkers for the detection of parenchymal brain metastasis: Current progress and future perspectives. Neurooncol Adv. 2026;8(1):vdag142. doi: 10.1093/noajnl/vdag142 42394645 PMC13326755

[34] Gupta VK, Cortese BD, Talwar R. Cost-effectiveness of serum, urine, and tissue-based prostate cancer biomarkers. Curr Opin Urol. 2025;35(4):412–7. doi: 10.1097/MOU.0000000000001293 40292531

[35] Lv L, Zhang W, Sun L, Zhao A, Zhang Y, Wang L, et al. Gene co-expression network analysis to identify critical modules and candidate genes of drought-resistance in wheat. PLoS One. 2020;15(8):e0236186. doi: 10.1371/journal.pone.0236186 32866164 PMC7458298

[36] Sánchez-Baizán N, Ribas L, Piferrer F. Improved biomarker discovery through a plot twist in transcriptomic data analysis. BMC Biol. 2022;20(1):208. doi: 10.1186/s12915-022-01398-w 36153614 PMC9509653

[37] Abiodun OA, Ola MS. Role of brain renin angiotensin system in neurodegeneration: An update. Saudi J Biol Sci. 2020;27(3):905–12. doi: 10.1016/j.sjbs.2020.01.026 32127770 PMC7042626

[38] Nuanpirom J, Charoensawan V. Multimodal bioinformatic analyses of genome-scale expression beyond gene-centric differential expression. Brief Bioinform. 2026;27(2):bbag152. doi: 10.1093/bib/bbag152 41978384 PMC13076944

[39] Smith J, Dobariya P, Kadam TR, More SS. Role of angiotensin-converting enzyme in cognitive aging: evidence from preclinical and clinical studies. Frontiers in Drug Discovery. 2026;6. doi: 10.3389/fddsv.2026.1855021 42272938 PMC13249463

[40] Arezoumandan S, Xie SX, Cousins KAQ, Mechanic-Hamilton DJ, Peterson CS, Huang CY, et al. Regional distribution and maturation of tau pathology among phenotypic variants of Alzheimer’s disease. Acta Neuropathol. 2022;144(6):1103–16. doi: 10.1007/s00401-022-02472-x 35871112 PMC9936795

[41] Shapiro DD, Lozar T, Cheng L, Xie E, Laklouk I, Lee MH, et al. Non-Metastatic Clear Cell Renal Cell Carcinoma Immune Cell Infiltration Heterogeneity and Prognostic Ability in Patients Following Surgery. Cancers (Basel). 2024;16(3):478. doi: 10.3390/cancers16030478 38339231 PMC10854750

[42] Toufiq M, Roelands J, Alfaki M, Syed Ahamed Kabeer B, Saadaoui M, Lakshmanan AP, et al. Annexin A3 in sepsis: novel perspectives from an exploration of public transcriptome data. Immunology. 2020;161(4):291–302. doi: 10.1111/imm.13239 32682335 PMC7692248

[43] Deng S, Xie H, Xie B. Cell-based regenerative and rejuvenation strategies for treating neurodegenerative diseases. Stem Cell Res Ther. 2025;16(1):167. doi: 10.1186/s13287-025-04285-7 40189500 PMC11974143

[44] Millot P, San C, Bennana E, Porte B, Vignal N, Hugon J, et al. STAT3 inhibition protects against neuroinflammation and BACE1 upregulation induced by systemic inflammation. Immunol Lett. 2020;228:129–34. doi: 10.1016/j.imlet.2020.10.004 33096140

