# Supplementary material for: Unraveling the molecular Nexus of Alzheimer’s Disease and HIV encephalitis: The role of cellular senescence and transcriptional regulation
Source: PLoS One. 2026 Aug 13;21(8):e0355548. doi: 10.1371/journal.pone.0355548 (PMC13472368; doi:10.1371/journal.pone.0355548)
Supplement: Supplementary file 1 [file pone.0355548.s003.docx]

Dear Editors and Reviewers,

We sincerely apologize for the delayed revision of our manuscript. We fully appreciate the valuable time and effort you have dedicated to reviewing our work, and we have carefully addressed all your insightful comments and suggestions. The extensive revisions were conducted to ensure the highest quality of presentation, reflecting our deep respect for your expertise and guidance. We are truly grateful for the opportunity to improve our manuscript and for the generous time provided for revisions.

_________________________

**### Response to Reviewer #1**

Thank you for your thoughtful review and constructive feedback, which have been instrumental in strengthening our manuscript. Your recognition of the study’s potential and detailed suggestions have guided our revisions effectively.

**Comment 1: Line 57 – The citation does not support the sentence, as the cited paper focuses on HAND rather than HIVE.**

**Response 1:** We appreciate this important observation. To address this, we have standardized the terminology throughout the revised manuscript by revising all references to "HIV encephalitis" to "HIV-associated neurocognitive disorders (HAND)" to align with the cited literature, ensuring the citation now appropriately supports the content.

**Comment 2: Lines 95–102 – This paragraph, including the figure, should be moved to the Results section.**

**Response 2:** Thank you for this organizational suggestion. We have relocated the paragraph describing the study workflow to the end of the revised manuscript, following the References, where all figure legends are now consolidated. This improves the logical flow by presenting the methodological overview within the results framework.

**Comment 3: Line 106 – Remove the extra space after the parentheses.**

**Response 3:** We appreciate catching this formatting error. We have carefully reviewed the entire revised manuscript and corrected all instances of extra spaces after parentheses to ensure consistent formatting.

**Comment 4: Figure 1 – Only two panels have reference letters; all plots should have letters, and legends should be updated for clarity.**

**Response 4:** Thank you for highlighting this inconsistency. We have revised Figure 1 in the revised manuscript by adding reference letters to all subpanels and updated the figure legend to clearly correspond with each panel, enhancing readability and clarity.

**Comment 5: Line 118 – Include references for the datasets used.**

**Response 5:** We agree with this suggestion. We have added the appropriate references for all datasets mentioned at their first occurrence in the revised manuscript to ensure traceability and reproducibility.

**Comment 6: Line 124 – Missing reference to the dataset.**

**Response 6:** Thank you for noting this omission. We have added the reference for the dataset at its first mention in the revised manuscript, ensuring all data sources are properly documented.

**Comment 7: Line 144 – Missing supporting reference.**

**Response 7:** We appreciate this reminder. A supporting reference for the bioinformatic method described has been added in the revised manuscript to validate the analytical approach.

**Comment 8: Line 153 – Missing reference for the dataset.**

**Response 8:** To address this, we have included the reference for the dataset at its first mention in the revised manuscript, ensuring all data sources are fully documented.

**Comment 9: Line 181 – Missing reference for GSE48350; explain the rationale for analyzing different datasets and clarify the "Samylicus" model with references and experimental details.**

**Response 9:** Thank you for this comprehensive comment. We have added the reference for GSE48350 at its first mention in the revised manuscript. Additionally, we have clarified the rationale for using multiple datasets in Lines 292–294 of the revised manuscript: different datasets were employed to enable internal validation (using primary datasets) and external validation (using independent cohorts), strengthening the robustness of our findings. The ambiguous term "Samylicus model" has been removed, and we have provided detailed descriptions of the experimental design of GSE48350, including sample characteristics and tissue sources, with corresponding references in the Methods section.

**Comment 10: Line 184 – Verify these numbers and report the source.**

**Response 10:** We have verified the numbers and added a clear citation to the original dataset publication as their source in the revised manuscript, ensuring transparency.

**Comment 11: Line 208 – Explain why GSE37263 was not included in this analysis.**

**Response 11:** We appreciate the need for clarification. GSE37263 was used as an internal dataset for initial discovery analyses. To validate our findings independently, we excluded it from subsequent validation steps in the revised manuscript and instead used external datasets. This approach ensures the reliability of our conclusions through cross-cohort verification.

**Comment 12: Line 216 – Clarify the rationale for "minimum of three samples," including power analysis, exclusion criteria, or why not all data were used.**

**Response 12:** Thank you for this important question. Upon re-evaluation, we recognized that using the full dataset (rather than a subset) would enhance statistical power. Thus, in the revised manuscript, we have analyzed the entire GSE48350 dataset, and the mention of "minimum of three samples" has been removed.

**Comment 13: Line 233 – Cite the supporting reference for GSE157829.**

**Response 13:** After careful revision, we decided to remove the single-cell analysis component from the revised manuscript to streamline focus. Consequently, GSE157829 is no longer used, and this reference is no longer necessary.

**Comment 14: General comment – Include a table summarizing all datasets, analysis types, references, and sample sizes for clarity.**

**Response 14:** We fully agree with this suggestion to enhance transparency. In the revised manuscript, we have integrated a comprehensive overview of all datasets into Figure 1 , which visually presents dataset identifiers, disease types, and tissue source. Additionally, detailed descriptions of each dataset, including references and sample counts, are provided in the Methods section at their first mention.

**Comment 15: Lines 320–322 – These should be in the Discussion, not Results.**

**Response 15:** Thank you for this structural feedback. We have revised the Results section in the revised manuscript to focus solely on presenting findings from the machine learning analysis of key genes, removing speculative interpretations. Contextual discussions have been relocated to the Discussion section.

**Comment 16: Lines 424–441 – These speculations are more suitable for the Discussion and require supporting literature.**

**Response 16:** To address this, we have removed the immune infiltration analysis section from the revised manuscript to refine focus. In the remaining Results sections, we have ensured that only empirical findings are presented, with all interpretive content moved to the Discussion section and supported by relevant literature.

**Comment 17: Line 442 – Focus on describing Figure 3 data and move speculation to the Discussion.**

**Response 17:** We appreciate this guidance. The immune checkpoint analysis (previously Figure 3) has been removed from the revised manuscript. For all remaining results, we have strictly focused on data description in the Results section, with interpretations reserved for the Discussion.

**Comment 18: Line 492 – Clarify the "Samylicus" model with standard nomenclature, sample size, methods, or references; if unpublished, describe tissue preparation.**

**Response 18:** As noted earlier, the ambiguous term "Samylicus model" has been removed from the revised manuscript. All datasets used are publicly available and properly cited, with detailed descriptions of sample characteristics and experimental methods provided in the Methods section.

**Comment 19: Line 750 – Provide in vitro validation of docking analysis, as docking alone does not demonstrate functional effects.**

**Response 19:** We acknowledge this limitation. To ensure the manuscript focuses on well-supported findings, we have removed the molecular docking analysis section from the revised manuscript, as in vitro validation was not available to confirm functional relevance.

_________________________

**### Response to Reviewer #2**

Thank you for your detailed review and invaluable suggestions to improve the manuscript’s clarity and organization. Your insights on structure and readability have significantly enhanced our presentation.

**Comment 1: Introduction – Lack of rationale for selected parameters, their definitions, and relevance to AD/HIVE; revise to include these details.**

**Response 1:** We agree with the need to strengthen this section. In the revised Introduction, we have added a comprehensive discussion explaining the rationale for focusing on transcriptional regulators and cellular senescence, including their known roles in AD pathogenesis, HAND mechanisms, and shared neuroinflammatory pathways. This provides clear context for their selection and relevance.

**Comment 2: Figure 1 legend – Relocate to the end with other figure legends, not in the Introduction.**

**Response 2:** Thank you for this organizational suggestion. The legend for Figure 1 has been moved to the end of the revised manuscript, following the References, where all figure legends are now consolidated.

**Comment 3: Materials and Methods – Written like a Results section; focus solely on methodologies without presenting results or referencing tables.**

**Response 3:** We have thoroughly revised the Materials and Methods section in the revised manuscript to remove all results-related content and table references. It now exclusively describes analytical pipelines, software tools, and dataset characteristics, adhering to methodological reporting standards.

**Comment 4: Line 142 – "Respectively" is unnecessary or should be repositioned if referring to bioinformatics packages for specific diseases.**

**Response 4:** We appreciate catching this ambiguity. The term "respectively" has been removed, and the sentence has been rephrased for clarity in the revised manuscript, specifying the application of each bioinformatics tool without redundant wording.

**Comment 5: Datasets – Reference five datasets across Materials and Methods; consolidate under a dedicated subsection.**

**Response 5:** This is an excellent suggestion. In the revised manuscript, datasets are introduced in a dedicated subsection within Materials and Methods, providing a centralized overview. Additionally, a visual summary of all datasets (including identifiers, disease types, and tissue sources) is presented in Figure 1, with detailed descriptions and references provided in the Methods section at their first mention.

**Comment 6: Figure 2 – Justify why datasets like GSE48350 are not included in panels A–C.**

**Response 6:** We have clarified this in the revised manuscript. Panels A–C of Figure 2 use internal datasets (GSE37263, GSE3489) for initial discovery of differentially expressed genes. GSE48350 was reserved for external validation, presented in Figure 4, to independently confirm these findings. This separation ensures robust validation of discovery results.

**Comment 7: Data interpretation – Discrepancies in gene expression patterns between figures (e.g., Figure 2A vs. 2F/4A-B; Figure 2B/2E vs. 3A-D); explain these differences.**

**Response 7:** Thank you for highlighting these inconsistencies. To resolve this, we have simplified Figure 2 in the revised manuscript by retaining only volcano plots (removing bean plots) to present differential expression results uniformly. All gene expression patterns are now consistent across figures, with clear labels indicating dataset sources to avoid confusion.

**Comment 8: Figure legends placement – Group all after References, not dispersed in Results.**

**Response 8:** We have implemented this suggestion. All figure legends are now consolidated at the end of the revised manuscript, following the References, for improved readability.

**Comment 9: Figure 3 – Specify the dataset used and explain the bar at the top of PDCDILG2 in 3K.**

**Response 9:** The immune checkpoint analysis (previously Figure 3K) has been removed from the revised manuscript to streamline focus. For remaining figures, dataset sources are clearly indicated in both figure legends and corresponding Results text.

**Comment 10: Consistent colors for groups across figures (e.g., green for Ctrl in Fig 2 vs. AD in Fig 5).**

**Response 10:** We appreciate this attention to detail. In the revised manuscript, we have standardized color coding across all figures, enhancing visual clarity.

**Comment 11: Figure 7A – Restore original proportions, not stretched.**

**Response 11:** As noted in Response 19 to Reviewer #1, the molecular docking analysis (previously Figure 7) has been removed from the revised manuscript, making this adjustment unnecessary.

_________________________

**### Additional Modifications and Clarifications**

In addition to addressing the reviewers’ comments, we have made the following important revisions:

1. **Author Affiliation Update**: Due to a change in workplace, the first author Hao Zhang’s affiliation has been updated to: *Geriatrics Center, Wuxi Second Geriatric Hospital, Wuxi, Jiangsu Province, China* (previously listed with two affiliations).

2. **Funding Statement**: We have updated the funding disclosure in the revised manuscript to include all supporting grants and clarified the funders’ role:

"This study was supported by the “Wuxi Science and Technology Development Fund (Award Number: Y20232005)”, “2025 Yangzhou University Provincial Key Lab Open Project for Integrative Chinese-Western Geriatric Disease Prevention & Treatment (Award Number: 202528)”, and “2025 Wuxi Aging Research Project (Award Number: WXLN25-A-24)” – all awarded to Hao Zhang. These funds enabled the research: the Wuxi Science and Technology Development Fund provided infrastructure support, including computational resources and database access. The Yangzhou University project funded molecular mechanism exploration, supporting gene expression and pathway analyses. The Wuxi Aging Research Project contributed to diagnostic model development and validation, supporting computational resources for optimization. "

3. **Code Sharing**: In accordance with PLOS ONE guidelines, we have added a statement in the Data Availability section of the revised manuscript indicating that all data underlying this study are fully available without restrictions.

4. **Regarding Figure Submission and PACE Access Issue**: We attempted to access the PACE website (https://pacev2.apexcovantage.com/) to verify figure formatting but encountered technical difficulties accessing the platform. However, we have carefully revised and optimized all figures in the revised manuscript to ensure they meet PLOS ONE’s style requirements, including consistent formatting, resolution, and labeling. We are happy to make further adjustments if needed.

_________________________

We have submitted two versions of the revised manuscript: a clean version and a marked-up version with changes highlighted for your convenience.

Once again, we express our deepest gratitude to both reviewers and editors for your invaluable feedback, patience, and guidance. Your expertise has significantly improved the quality and rigor of our work. We hope the revised manuscript now meets PLOS ONE’s publication criteria.

Thank you for your time and consideration.

Best regards,

Wei Lu
