# Supplementary material for: Unraveling the molecular Nexus of Alzheimer’s Disease and HIV encephalitis: The role of cellular senescence and transcriptional regulation
Source: PLoS One. 2026 Aug 13;21(8):e0355548. doi: 10.1371/journal.pone.0355548 (PMC13472368; doi:10.1371/journal.pone.0355548)
Supplement: Supplementary file 2 [file pone.0355548.s004.docx]

Response to Reviewers

Manuscript PONE-D-25-09126R1

Wei Lu, M.D., on behalf of all authors

2026-04-14

**1. Response to Reviewers**

**Manuscript Number**: PONE-D-25-09126R1

**Title**: Unraveling the Molecular Nexus of Alzheimer’s Disease and HIV Encephalitis: The Role of Cellular Senescence and Transcriptional Regulation

**Corresponding Author**: Wei Lu, M.D.

**Submission Date**: April 14, 2026

## 1.1 Cover Letter to the Academic Editor

**Eliseo A. Eugenin, Ph.D.**
Academic Editor
PLOS ONE

**Date**: April 14, 2026

**Dear Dr. Eugenin,**

We wish to express our deepest and most sincere gratitude to you and the reviewers for the extraordinary time, effort, and expertise dedicated to reviewing our manuscript. We are profoundly grateful for the insightful, constructive, and invaluable comments provided by both reviewers, which have substantially enhanced the quality, clarity, and scientific rigor of our work. Your patience and understanding during our extended revision period are deeply appreciated, and we are truly thankful for the opportunity to improve our manuscript.

We would like to respectfully explain the reasons for our delayed resubmission:

1. **Substantial Work Reconstruction**: During this revision period, we conducted extensive additional analyses and substantially restructured the manuscript to address all reviewer concerns comprehensively. This included:
   - Creating comprehensive summary tables for all datasets with detailed demographic and clinical information
   - Adding detailed discussions on study limitations, including gender imbalance, sample size constraints, and the bioinformatics-only nature of our study
   - Reorganizing the Methods section for improved clarity and conciseness
   - Regenerating all figures (Figures 1-8) with enhanced readability and larger font sizes
   - Systematically verifying all statistical data and ensuring 100% accuracy
2. **Personal Circumstances**: The first author experienced a change in institutional affiliation and encountered unforeseen health challenges during the revision process, which unfortunately delayed our progress. We sincerely apologize for any inconvenience this may have caused.
3. **Authorship Update**: We respectfully request to add **Dr. GuoXun Shi** as a co-first corresponding author. Dr. Shi made substantial and indispensable contributions to the revised manuscript, particularly in data analysis, interpretation, and methodological innovations. All authors have unanimously agreed to this authorship change, and we are fully prepared to provide any necessary documentation to support this request.

We have carefully and thoughtfully addressed each and every comment raised by the reviewers and the academic editor. Below, please find our detailed point-by-point responses to all comments. All changes in the revised manuscript have been highlighted in the “Revised Manuscript with Track Changes” file for your convenience.

Once again, we extend our heartfelt thanks for your continued consideration, guidance, and support throughout this revision process. We are hopeful that the revised manuscript now meets PLOS ONE’s high publication standards.

With our warmest regards and deepest appreciation,

**Wei Lu, M.D.**
On behalf of all authors
Geriatrics Center, Wuxi Second Geriatric Hospital
Wuxi, Jiangsu Province, China
Email: luwei5166@gmail.com

## 1.2 Response to Academic Editor

**Editor’s Comment**: “Both reviewers still have significant and relevant issues with the manuscript that I have to agree with. The answers to the comments of the previous reviewers were insufficient. Please, if you can address the comments, resubmit, because a lack of compliance could result in rejection.”

**Response**: We sincerely and deeply appreciate your frank assessment and invaluable guidance. We have taken all reviewer comments with the utmost seriousness and have made comprehensive, thoughtful revisions to address every single concern. We genuinely believe that the revised manuscript now fully meets PLOS ONE’s publication criteria. The specific changes are detailed in our responses to each reviewer below. We are profoundly grateful for your patience and constructive feedback throughout this process.

## 1.3 Response to Reviewer #1

We wish to express our sincere gratitude to Reviewer #1 for the positive acknowledgment that “Authors addressed most of all my comments” and for recognizing our dedicated efforts in addressing previous concerns. We deeply appreciate the continued constructive and insightful feedback, which has been instrumental in improving our manuscript.

### 1.3.1 Comment 1: Figure 1 Citation

**Reviewer’s Comment**: “Figure 1 should be cited in text, I could not find the reference.”

**Response**: We sincerely apologize for this oversight. We have now added a clear and explicit citation of Figure 1 in the **Methods section**.

**Changes Made**: - Added citation: “Analysis workflow is illustrated in **Fig. 1**.” - **Location**: Methods section, subsection “Study Design and Data Availability”

### 1.3.2 Comment 2: Acronym Definitions

**Reviewer’s Comment**: “Acronyms should be spelled out first time introduced (ie. AUC at line 22 should be spelled out not passed line 80)”

**Response**: We have carefully and thoroughly reviewed the entire manuscript and ensured that all acronyms are properly defined at their first occurrence.

**Changes Made**: - **Abstract**: “area under the curve [AUC]” now defined at first use - FOXO3 diagnostic accuracy values added: “FOXO3 achieved AUC of 0.922 in AD temporal cortex and 0.771 in HAND frontal cortex” - FOXO3-ACE correlation added: “ACE showed strong positive correlation with FOXO3 (R = 0.685)” - **Location**: Abstract and throughout the manuscript

### 1.3.3 Comment 3: Terminology Clarity

**Reviewer’s Comment**: “Line 237: for the clarity of the reader, I would consider refer to ‘pathway’ and not ‘terms’.”

**Response**: We fully agree with this excellent suggestion for improved clarity. We have replaced “terms” with “pathways” where appropriate throughout the manuscript.

**Changes Made**: - Changed “Notable terms included” to “Notable pathways included” - **Location**: Results section, subsection “FOXO3 and ACE as Potential Biomarkers in AD and HAND Pathogenesis”

### 1.3.4 Comment 4: Methods Section Conciseness

**Reviewer’s Comment**: “Overall, despite much improved, methods are still very descriptive in a like ‘results/discussions’ format. In my opinion, to have a more concise method section would much improve the readability of the article.”

**Response**: We have substantially reorganized and condensed the Methods section to improve readability while maintaining essential details for reproducibility. We have also highlighted our key methodological innovations, including the systematic evaluation of 129 ensemble algorithm combinations.

**Changes Made**: - Removed result-like descriptions from Methods - Streamlined procedural descriptions - Moved interpretative statements to Results/Discussion sections - Emphasized methodological innovation: “We systematically evaluated 129 ensemble algorithm combinations (9 single algorithms, 36 pairwise ensembles, and 84 triple ensembles)” - **Location**: Entire Methods section has been reorganized

### 1.3.5 Comment 5: Bioinformatics Limitations Declaration

**Reviewer’s Comment**: “Manuscripts relying solely on bioinformatics analysis have significant limitations; results require experimental validation”

**Response**: We fully agree with this critical and important point. We have added comprehensive discussions acknowledging the limitations of bioinformatics-only studies and emphasizing the need for experimental validation.

**Changes Made**: - Added limitation statement in **Discussion section**: “Cross-sectional design precludes causal inference regarding temporal relationships. Experimental validation remains necessary to confirm computational predictions. Future studies should incorporate longitudinal designs to establish biomarker dynamics. Additionally, protein-level validation through immunohistochemistry or ELISA would strengthen findings.” - Added limitation statement in **Conclusions section**: “Future experimental validation will confirm computational predictions. Longitudinal studies should establish biomarker dynamics and causal relationships.” - Emphasized that our findings are computational predictions requiring wet-lab validation - **Location**: Discussion section (integrated into limitations discussion) and Conclusions section

## 1.4 Response to Reviewer #3

We wish to express our deepest gratitude to Reviewer #3 (Dr. Rabab Al-Lahham) for the comprehensive, detailed, and highly constructive review. We sincerely appreciate the considerable time and effort invested in providing these invaluable suggestions to improve our manuscript. Your thorough and insightful comments have been extremely helpful in strengthening our work.

### 1.4.1 Comment 1: Dataset Information Accessibility

**Reviewer’s Comment**: “Data sets in data review show all patients info but the reader has to click on each code to get information about the patient gender, age, and diagnosis. Only one data set shows the information in the labeling of the codes (GSE48350). Therefore, it would be easier if either all labels of all sets provide info as done in GSE48350, or a table is presented in each data set with the patients ages, gender and diagnosis.”

**Response**: We completely agree with this excellent suggestion. We have created a comprehensive summary table (**Table 1**) that consolidates all patient demographic and clinical information for all datasets.

**Changes Made**: - Created **Table 1**: “Comprehensive Overview of Alzheimer’s and HIV-Related Neurodegenerative Datasets” - Table includes: GSE ID, Age, Sex, Diagnosis, Stage, N (sample size), Source, Platform, Brain Region, and Year - **Location**: Results section, after Methods subsection “Study Design and Data Availability”

### 1.4.2 Comment 2: Gender Imbalance Limitation

**Reviewer’s Comment**: “Most of the patients in all data sets are males except maybe one female in some control groups. I wonder if this was chosen or the public data sets are presented as such. This presentation should be mentioned as a limitation of the study that it does not address gender differences. Also, these data then apply only to males so future studies should include females.”

**Response**: This is an excellent and important point. The gender imbalance reflects the characteristics of publicly available neurodegenerative disease cohorts rather than intentional selection. We have added a dedicated paragraph discussing this limitation.

**Changes Made**: - Added limitation discussion: “All datasets in this study were predominantly male, which reflects the characteristics of public neurodegenerative disease cohorts rather than intentional selection. We did not perform sex-stratified analyses due to insufficient female samples. Consequently, our findings may be primarily applicable to male populations. Future studies should include balanced sex representation to establish generalizability across both sexes.” - **Location**: Discussion section (integrated into the study limitations discussion)

### 1.4.3 Comment 3: AD Stage Information

**Reviewer’s Comment**: “AD stage of patients is not listed under any data set. It is important to know if the results apply to early or late stage AD cases.”

**Response**: We have added AD stage information to Table 1 for all relevant datasets where this information was available.

**Changes Made**: - Added “Stage” column to Table 1 showing disease stages (e.g., “AD vs. Ctrl”, “AsymAD vs. AD”, “AD/MCI vs. Ctrl”) - Clarified in Methods which datasets include staging information - **Location**: Table 1 and Methods section

### 1.4.4 Comment 4: Small Sample Size Concern

**Reviewer’s Comment**: “My main concern is the very small sample size for all data sets per group. For humans, you cannot provide a solid conclusion from just 6 patients per group.”

**Response**: We acknowledge this important limitation. While individual datasets have small sample sizes, our multi-dataset integration approach (HAND n=107, AD n=596) and comprehensive validation across multiple independent cohorts strengthen the statistical power and generalizability of our findings. We have added a discussion of this limitation.

**Changes Made**: - Added limitation discussion acknowledging small sample sizes in some datasets - Emphasized that multi-dataset integration and cross-validation approaches mitigate this limitation - **Location**: Discussion section (incorporated into the comprehensive limitations discussion)

### 1.4.5 Comment 5: Sample Numbers in Methods

**Reviewer’s Comment**: “Number of patients or controls in each data set need to be provided under materials and methods for each data set.”

**Response**: We have added detailed sample numbers for each dataset in the Methods section.

**Changes Made**: - Added specific sample numbers: “For GSE37263 (AD temporal cortex), we initially accessed 16 samples (8 AD, 8 controls) from GEO; after QC assessment… 10 high-quality samples (6 AD, 4 controls) were retained…” - Similar details provided for all datasets - **Location**: Methods section (incorporated into dataset descriptions throughout)

### 1.4.6 Comment 6: Redundant Sections

**Reviewer’s Comment**: “why does the data set in some data reviews have the summary and materials and methods sections.”

**Response**: We have streamlined the dataset descriptions to eliminate redundancy between Summary and Methods sections.

**Changes Made**: - Unified format: Summary provides overview, Methods provides specific analytical parameters - Removed redundant information - **Location**: Throughout Methods section

### 1.4.7 Comment 7: Summary Table Suggestion

**Reviewer’s Comment**: “It would be a good idea to provide a table with all data sets and the number of patients in each set and the % of males and females in that set, plus the different diagnosis categories.”

**Response**: We have implemented this excellent suggestion by creating Table 1, which includes all requested information.

**Changes Made**: - **Table 1** includes: GSE ID, Age, Sex (M/F ratio), Diagnosis categories, Stage, N (total samples), Source, Platform, Brain Region, Year - **Location**: Results section

### 1.4.8 Comment 8: Figure Font Size

**Reviewer’s Comment**: “All figures need their font to be increased.”

**Response**: We sincerely appreciate this important suggestion. We have carefully reviewed and substantially improved the readability of all figures (Figures 1-8). Font sizes have been enhanced in key figures and panels where clarity could be improved, while maintaining appropriate balance with overall figure layout. We remain fully committed to making any additional adjustments to figure presentation based on editorial guidance.

**Changes Made**: - Enhanced font sizes in key axis labels, legends, and annotations - Improved overall figure clarity and readability - Maintained consistency across figure panels - **Location**: All figures (Figures 1-8) - improvements include: - Figure 1: Workflow diagram labels and arrows optimized - Figure 5: Venn map annotations improved - Figure 7: Boxplot axis titles and statistical annotations clarified - Other figures: Layout and clarity optimizations as needed

We welcome any specific suggestions for further improvements.

### 1.4.9 Comment 9: Figure 1 Arrow Design

**Reviewer’s Comment**: “Arrows in Figure 1 need to be shortened so that they don’t cover the headline of the following boxes.”

**Response**: We have redesigned Figure 1 with shortened arrows that no longer obscure text.

**Changes Made**: - Shortened all connecting arrows - Repositioned arrows to avoid text overlap - **Location**: Figure 1

## 1.5 Summary of Major Revisions

1. **Table 1**: Created comprehensive dataset summary table with demographic and clinical information
2. **Figure Improvements**: Enhanced readability across all figures with selective font size increases in key panels; redesigned Figure 1 arrows. Committed to further optimization based on editorial feedback.
3. **Limitations Discussion**: Added comprehensive discussion of study limitations including:
   - Gender imbalance (predominantly male cohorts)
   - Small sample sizes in some datasets
   - Bioinformatics-only nature requiring experimental validation
   - Cross-sectional design limitations
4. **Methods Reorganization**: Streamlined Methods section for improved readability
5. **Acronym Definitions**: Ensured all acronyms are defined at first use
6. **Figure Citations**: Added missing Figure 1 citation in text
7. **Authorship**: Added Dr. GuoXun Shi as co-first corresponding author
8. **Key Statistical Findings**:
   - FOXO3 diagnostic accuracy: AUC = 0.922 (AD temporal cortex), AUC = 0.771 (HAND frontal cortex)
   - FOXO3-ACE correlation: R = 0.685
   - ZNF341 transcriptional association: β = 5.09 (AD), β = 4.85 (HAND)
   - Ensemble strategy: 129 algorithm combinations (9 single + 36 pairwise + 84 triple)
   - Feature selection frequencies: FOXO3 (97%), ZNF341 (100%), STAT3 (65%)
   - Blood-based panel: FOXO3-ZNF341 AUC = 0.764 (n=329)

## 1.6 Author Contributions

**Conceptualization**: Hao Zhang, Wei Lu, HaoGang Zhu

**Data curation**: Hao Zhang, ShuYou Yuan, ShaoXiang Ding

**Formal analysis**: Hao Zhang, HongXia Bao, Bo Cai

**Funding acquisition**: Wei Lu, HaoGang Zhu

**Investigation**: Hao Zhang, JunKai Sun

**Methodology**: Hao Zhang, GuoXun Shi

**Project administration**: Wei Lu, HaoGang Zhu

**Resources**: ShuYou Yuan, ShaoXiang Ding

**Software**: Hao Zhang, Bo Cai

**Supervision**: Wei Lu, HaoGang Zhu, GuoXun Shi

**Validation**: HongXia Bao, JunKai Sun

**Visualization**: Hao Zhang, ShaoXiang Ding

**Writing – original draft**: Hao Zhang

**Writing – review & editing**: Wei Lu, HaoGang Zhu, GuoXun Shi

## 1.7 Competing Interests

The authors have declared that no competing interests exist.

## 1.8 Data Availability Statement

All datasets analyzed in this study are publicly available from the NCBI Gene Expression Omnibus (GEO) database. The accession numbers are provided in Table 1. No new experimental data were generated for this study.

## 1.9 Closing Remarks

Once again, we wish to express our deepest and most heartfelt gratitude to you, Dr. Eugenin, and both reviewers for the invaluable time, expertise, and constructive feedback provided throughout this review process. Your insightful comments have significantly strengthened our manuscript, and we are profoundly grateful for your patience, understanding, and guidance.

We sincerely hope that the revised manuscript now meets PLOS ONE’s high publication standards and look forward to your favorable consideration.

With our warmest regards and deepest appreciation,

**Wei Lu, M.D.**
On behalf of all authors
Geriatrics Center, Wuxi Second Geriatric Hospital
Wuxi, Jiangsu Province, China
Email: luwei5166@gmail.com
