# Supplementary material for: Unraveling the molecular Nexus of Alzheimer’s Disease and HIV encephalitis: The role of cellular senescence and transcriptional regulation
Source: PLoS One. 2026 Aug 13;21(8):e0355548. doi: 10.1371/journal.pone.0355548 (PMC13472368; doi:10.1371/journal.pone.0355548)
Supplement: Supplementary file 3 [file pone.0355548.s005.docx]

**Response to Reviewers**

**Manuscript Number:** PONE-D-25-09126R2

**Title:** Unraveling the Molecular Nexus of Alzheimer's Disease and HIV Encephalitis: The Role of Cellular Senescence and Transcriptional Regulation

────────────────────────────────────────────────────────────

Dear Editor and Reviewers,

We sincerely thank the Editor and both Reviewers for their thoughtful and constructive comments on our revised manuscript. These suggestions have helped us further improve the clarity, accuracy, and quality of our work. We have carefully addressed each point and made corresponding revisions to the manuscript. Below, we provide a point-by-point response detailing all changes made. All references to sections and figures correspond to the revised manuscript with tracked changes.

────────────────────────────────────────────────────────────

**Reviewer #1**

**Comment R1-1: Figure legends should contain more information**

Reviewer Comment: "Figure legends should contain more information. Figures are very dense and Figure legends should allow figures to be self explanatory. For example: Figure 1 legend should briefly describe the workflow."

Response: We thank the Reviewer for this valuable suggestion. We fully agree that figure legends should be sufficiently detailed to allow independent interpretation. We have revised all figure legends as follows:

Fig. 1: We have added a concise workflow description summarizing the sequential analytical steps (data acquisition, preprocessing, differential expression, machine learning, transcription factor inference, ensemble evaluation, and blood-based validation), with dataset annotations for each step.

Fig. 2–8: Each figure legend now includes: (i) the dataset accession number(s) used; (ii) a brief description of what each panel represents; (iii) the statistical test applied where relevant.

Specific additions: Fig. 2 now explicitly states which panels correspond to GSE37263 (AD) vs. GSE3489 (HAND). Fig. 4 distinguishes GSE48350 panels (A–E, J) from GSE122063 panels (F–I). Fig. 3, 5, 7, and 8 legends now include dataset identifiers.

*(Revised Manuscript: Figure Legends section)*

────────────────────────────────────────────────────────────

**Comment R1-2: Panels F and H look stretched**

Reviewer Comment: "Panels F and H look stretched. Quality should be fixed."

Response: We thank the Reviewer for identifying this quality issue. Upon inspection, we confirmed that Fig. 7 Panels F and H (nomograms for HAND and AD cohorts) were affected by incorrect aspect ratio during figure compilation. We have:

Re-exported both panels from source data at 600 DPI with correct aspect ratio.

Conducted a full quality check on all figure panels to confirm no similar issues exist elsewhere.

All figures will be submitted as high-resolution TIFF files meeting PLOS ONE requirements (≥300 DPI).

*(Revised Manuscript: Figure 7; Figure files will be updated in the final submission)*

────────────────────────────────────────────────────────────

**Reviewer #3**

**Comment R3-1a: Table 1 — "Elderly" should be replaced with specific age range**

Reviewer Comment: "Page 5, Table 1: 'age as elderly' needs to be changed with at least an age range (78.5 ± 9.2 years for AD temporal cortex, 52.3 ± 8.7 for HAND frontal cortex and 74.2 ± 7.8 for AD blood) like how was described above or elderly needs to be defined in the Table legend."

Response: We agree that "Elderly" is insufficiently specific. We have revised Table 1 as follows:

Replaced the "Age" column entries with the specific values already reported in the Methods section: GSE37263 (78.5 ± 9.2 years), GSE3489 (52.3 ± 8.7 years, verified from original manuscript Methods section, now in the table with a footnote marker[§]), GSE63060 (74.2 ± 7.8 years), GSE48350 (20–99 years, verified from the series matrix sample annotations).

For datasets where individual-level age data were not available in GEO records (GSE28160, GSE35864, GSE118553, GSE122063, GSE132903), we have marked the entry as "N/S" (Not Specified) with a footnote.

Added a table footnote explaining the source of age data and acknowledging this limitation.

**Limitation acknowledged:** We recognize that age information is unavailable for several datasets. This reflects a limitation of publicly available transcriptomic datasets rather than an oversight in our analysis. We have noted this in both Table 1 and the Discussion section.

*(Revised Manuscript: Table 1, with revised "Age" column and additional footnotes)*

────────────────────────────────────────────────────────────

**Comment R3-1b: Table 1 — "M/F" should be replaced with specific numbers of males and females**

Reviewer Comment: "Write the number of males and females rather than M/F or the % of males."

Response: We thank the Reviewer for this specification. Upon further inspection of the original GEO records, we found that per-sample sex data were not uniformly available across all datasets. We have revised Table 1 as follows:

GSE63060: 191M / 138F (58% male, from 329 samples) — sex data available from the AddNeuroMed cohort.

GSE48350: 124M / 129F (verified from the series matrix sample annotations, 253 samples across 4 brain regions).

For all other datasets (GSE3489, GSE28160, GSE35864, GSE37263, GSE118553, GSE122063, GSE132903), per-sample sex data were not accessible in the original GEO records. The entry is marked "N/S" (Not Specified) with a footnote.

**Correction note:** In the previous revision, we reported GSE37263 as 7M/3F based on the subset of 10 post-QC samples. However, upon further verification, we found that per-sample sex information for the 16 original samples (8 AD, 8 Ctrl) was not uniformly recorded in the GEO deposit. To maintain strict data integrity, we have revised this entry to "N/S" in the updated Table 1.

**Limitation acknowledged:** We recognize that sex information is unavailable for most datasets. This reflects a common limitation of publicly available transcriptomic datasets, particularly postmortem brain tissue studies where demographic annotation is often incomplete. We have noted this in both Table 1 and the Discussion section.

*(Revised Manuscript: Table 1, "Sex (M/F)" column updated)*

────────────────────────────────────────────────────────────

**Comment R3-1c: Table 1 — N should be the final QC number**

Reviewer Comment: "N should be the finalized number used in the study not the initial one chosen, the original numbers can be explained under the method section. If N at the top of the table is for the initial selection before the QC assessment, it needs to be defined as such."

Response: We agree. We have revised Table 1 to include two columns: "Total N (final)" for samples included after QC (as specified in Methods), and "Initial N" for the original cohort size before QC filtering. The Methods section already describes the QC process and sample selection criteria for GSE37263 (16→10), GSE3489 (28→12 selected), and GSE63060 (329 all passed QC). For transparency, initial N values are now explicitly listed alongside final N.

*(Revised Manuscript: Table 1, with new "Initial N" column; Methods section)*

────────────────────────────────────────────────────────────

**Comment R3-1d: Table 1 — per-group N should be specified**

Reviewer Comment: "The number per group needs to be specified rather than a total number for all cases in that data set. For example, GSE3489 17 AIDS pts (HIVE+ vs. HIVE-); GSE35864 24 subj (Ctrl, HIV, HAD, HIVE) needs to be specified with the number per group and remove the word subj. Same here GSE48350 AD vs. Ctrl and GSE63060 AD, MCI vs. Ctrl AD/MCI vs. Ctrl."

Response: We have revised Table 1 to include per-group sample sizes in the "Diagnosis (per-group N)" column, verified wherever possible from the original GEO SOFT files and series matrix annotations:

GSE3489: HIVE+ (8), HIVE- (6) — verified from SOFT file (14 unique individuals, each with A/B technical replicates; 28 total arrays)

GSE35864: Ctrl (6), HIV (6), HAND (7), HIVE (5) — verified from GEO record (24 subjects across 3 brain regions)

GSE37263: AD (6), Ctrl (4)

GSE63060: AD (145), MCI (80), Ctrl (104)— per-group N from raw GEO data; see footnote for AddNeuroMed classification details

GSE118553: Ctrl (27), AsymAD (33), AD (52)

GSE122063: VaD (8), AD (12), Ctrl (11)

GSE132903: AD (97), Ctrl (98)

GSE48350: AD (28), Ctrl (56) — verified from series matrix titles (84 unique individuals, 253 samples across 4 brain regions)

*(Revised Manuscript: Table 1, "Diagnosis (per-group N)" column)*

────────────────────────────────────────────────────────────

**Comment R3-1e: Table 1 — "HAD" should be "HAND"**

Reviewer Comment: "What is HAD in data set GSE35864? It should be HAND."

Response: We apologize for this terminology error. "HAD" (HIV-Associated Dementia) has been corrected to "HAND" (HIV-Associated Neurocognitive Disorders) throughout Table 1 and the entire manuscript, including all figure legends and the main text. We have performed a full-text audit to ensure consistent use of "HAND" throughout.

*(Revised Manuscript: Table 1, full-text HAD→HAND replacement)*

────────────────────────────────────────────────────────────

**Comment R3-1: Table 1 — Data Limitations Statement**

Response: We acknowledge that certain datasets (GSE3489, GSE28160, GSE35864) lack complete age, sex, and per-group sample size information in the original GEO records. This reflects the inherent limitations of publicly available transcriptomic datasets rather than an oversight in our analysis. We have noted these data gaps in Table 1 using "N/S" (Not Specified) and have added a comprehensive limitations statement in the Discussion section.

**Limitations acknowledged:** We recognize that the absence of individual-level demographic data for several HAND datasets limits our ability to perform age- and sex-stratified analyses. This is a common challenge in meta-analyses of public repository data. We have explicitly stated this limitation in the Discussion and recommend that future prospective studies include standardized clinical annotation to enable more granular subgroup analyses.

**Table optimization:** To balance comprehensiveness with readability, we have streamlined Table 1 from 10 columns to 7 columns by consolidating the "Platform" and "Initial N (Pre-QC)" columns into footnotes. This reduces visual clutter while maintaining all essential information required by the reviewer.

*(Revised Manuscript: Table 1, Page 5; Discussion — Limitations section)*

────────────────────────────────────────────────────────────

**Comment R3-1f: GSE48350 age range 20–99 yrs is a broad range**

Reviewer Comment: "GSE48350 20-99 yrs is a huge range."

Response: We acknowledge this concern. GSE48350, obtained from the Harvard Brain Tissue Resource Center, encompasses a wide age range reflecting both normal aging controls and AD cases. We have:

Added a footnote in Table 1 explaining: "GSE48350 spans an age range of 20–99 years, reflecting the heterogeneous donor population. Age-stratified analysis was not feasible due to limited per-stratum sample sizes. This is noted as a limitation."

Expanded the Limitations section in the Discussion to address the potential for age-related confounding.

Note that our primary machine learning models used 5-fold cross-validation, which mitigates overfitting to age-related expression patterns.

*(Revised Manuscript: Table 1 footnote; Discussion — Limitations)*

────────────────────────────────────────────────────────────

**Comment R3-1g: AD stage not listed for many datasets**

Reviewer Comment: "Again, AD stage for many datasets is not listed."

Response: We agree that AD stage information is valuable contextual data. We have added a "Disease Stage" column to Table 1. Where available in the original GEO records, staging information has been included (e.g., GSE118553: AsymAD vs. AD). For datasets where staging was not deposited, we have marked "N/S" (Not specified).

Regarding GSE48350 specifically, we note that MMSE scores are available for the AD samples (range 0–27, 80 samples from 28 individuals), but Braak neurofibrillary tangle staging was not deposited in the GEO record. We have therefore retained "N/S" for disease stage in this dataset. This limitation is acknowledged in the Discussion.

*(Revised Manuscript: Table 1, new "Disease Stage" column; Discussion — Limitations)*

────────────────────────────────────────────────────────────

**Comment R3-2: Figure 2C was done for GSE3489 — should it be HAND?**

Reviewer Comment: "Figure 2C; this was done for data set GSE3489, should it be HAND?"

Response: The Reviewer is correct. Fig. 2C presents the Venn diagram analysis of overlapping DEGs between AD (GSE37263), HAND (GSE3489), and cellular senescence genes. We have:

Revised the figure legend to explicitly state: "Venn diagram of overlapping DEGs among AD (GSE37263), HAND (GSE3489), and 279 cellular senescence-related genes."

Updated the corresponding Results text to clarify the HAND classification.

Ensured that all dataset-to-condition mappings are consistent throughout.

*(Revised Manuscript: Fig. 2C legend; Results section)*

────────────────────────────────────────────────────────────

**Comment R3-3: Figures should indicate dataset numbers**

Reviewer Comment: "Figure 2: the reader can get lost and has to go back and forth to check to which data set the analysis in the different figures is done. It is better to either write in the legend again or at the top of each figure the data set number the analysis was performed on. Same for all figures."

Response: We fully agree. We have systematically annotated all figures with dataset accession numbers:

In each figure legend, the dataset ID is now stated at the beginning (e.g., "Fig. 2. FOXO3 and ACE as potential biomarkers in AD and HAND (GSE37263 for AD panels; GSE3489 for HAND panels).")

In the Results text, each figure citation now includes the dataset ID in parentheses (e.g., "(Fig. 2A, GSE37263)").

Specific dataset assignments for multi-panel figures are clearly delineated (e.g., Fig. 4: GSE48350 for panels A–E, J; GSE122063 for panels F–I).

*(Revised Manuscript: All Figure legends; Results section throughout)*

────────────────────────────────────────────────────────────

**Comment R3-4: ACE shows no difference in Figs. 3E and 4J — how is it a biomarker?**

Reviewer Comment: "Figures 3E and 4J show no difference in ACE between controls and HAND or AD groups. Then how would it be considered a biomarker for HAND and AD?"

Response: We thank the Reviewer for this important question, which has prompted us to clarify our analytical framework. We have made the following revisions:

**1. Explicit acknowledgment in Results:** We have added sentences in the Results section explicitly stating that ACE showed no significant difference between groups in Fig. 3E (HAND) and Fig. 4J (AD).

**2. New Discussion paragraph:** We have added a dedicated subsection entitled "ACE as a Co-Expression Biomarker Rather Than a Differential Biomarker" in the Discussion. This section explains:

ACE was identified through its strong and consistent co-expression with FOXO3 across multiple independent datasets, not through differential expression analysis.

ACE showed Spearman R = 0.685 (p < 0.01) with FOXO3 in GSE37263 and was consistently ranked as a top feature in regression models predicting FOXO3 expression (Fig. 2Q, 3I, 4D).

ACE functions as a co-expression network biomarker — its value lies in its regulatory association with FOXO3 (implicating renin-angiotensin system involvement) rather than as an independent classifier.

The distinction between differential expression biomarkers and co-expression biomarkers is well established in transcriptomic studies.

In the ensemble diagnostic framework (Fig. 8), ACE alone showed low selection frequency across 129 algorithm combinations (Fig. 8H), consistent with limited independent diagnostic value. The primary diagnostic candidates remain FOXO3, ZNF341, and STAT3.

**3. Revised terminology in Results section:** We have changed the Results subsection title from "FOXO3 and ACE as Potential Biomarkers in AD and HAND Pathogenesis" to "FOXO3 as a Primary Biomarker and ACE as a Co-Expression Partner in AD and HAND Pathogenesis". This change more accurately reflects ACE's role in our analytical framework.

**4. Enhanced conceptual framework:** The new Discussion paragraph now includes:

A clear distinction between two types of biomarkers: differential expression biomarkers vs. co-expression biomarkers

Biological context: ACE's role in the renin-angiotensin system (RAS) and its mechanistic link to FOXO3-mediated cellular senescence pathways

Explicit statement that ACE's value lies in its role as a network hub reflecting FOXO3-associated regulatory activity, not as an independent classifier

*(Revised Manuscript: Results — title changed to clarify ACE's role; Discussion — expanded "ACE as a Co-Expression Biomarker" subsection with conceptual framework and biological interpretation)*

────────────────────────────────────────────────────────────

**Comment R3-5: Results section title contains logical inconsistency**

Reviewer Comment: "Result section: 'Key Genes FOXO3 and ACE Identified in GSE48350 with Diagnostic Potential in GSE122063 Datasets': all data in Figure 4 were for the set GSE48350 which is a hippocampus data set, then how can the different results be applied for cortex (frontal and temporal) and why the data set GSE122063 included in this result section title without being referred to."

Response: We sincerely thank the Reviewer for identifying this logical inconsistency. We have made the following corrections:

**1. Revised the Results subsection title:**

Original: "Key Genes FOXO3 and ACE Identified in GSE48350 with Diagnostic Potential in GSE122063 Datasets"

Revised: "Diagnostic Performance of FOXO3 and ACE Across Brain Regions: Hippocampal (GSE48350) and Cortical (GSE122063) Validation"

**2. Added introductory paragraph:** We now begin the section with a clear statement explaining the multi-region validation design: "To evaluate the regional consistency of biomarker performance, we analyzed FOXO3 and ACE in two independent AD datasets: GSE48350 (hippocampus) and GSE122063 (frontal and temporal cortex). This multi-region approach allows assessment of whether biomarker performance is consistent across different brain regions affected by AD."

**3. Clarified GSE122063 usage in the body text:** Fig. 4 panels are explicitly labeled: panels A–E and J correspond to GSE48350 (hippocampus), while panels F–I correspond to GSE122063 (frontal and temporal cortex). The text now accurately describes both cohorts with dataset IDs at each reference.

**4. Regional applicability acknowledged:** In the Discussion, we note that the hippocampus-derived signatures (GSE48350) show partial concordance with cortical signatures (GSE122063), and that regional transcriptomic differences warrant further investigation.

**5. New Discussion section added — "Multi-Region Validation: Scientific Rationale and Implications":** We added a dedicated subsection explaining the scientific rationale for multi-region validation:

The hippocampus is typically affected earlier in AD, while cortical regions show progressive involvement.

FOXO3 shows consistent diagnostic performance across hippocampus (AUC = 0.711) and cortex (AUC = 0.708), suggesting it captures a fundamental aspect of AD pathophysiology.

This regional consistency strengthens the generalizability of our findings and supports biomarker translation from post-mortem brain tissue to peripheral blood samples.

*(Revised Manuscript: Results section title and §4 body; Fig. 4 legend; Discussion — new subsection "Multi-Region Validation")*

────────────────────────────────────────────────────────────

**Additional Changes Made**

Beyond the specific reviewer comments, we have also:

Language polishing: The entire manuscript has been reviewed for clarity, grammar, and readability.

Full-text terminology audit: All instances of "HAD" → "HAND" corrected; "HIVE" vs "HAND" usage verified throughout.

Figure legends comprehensively updated: All legends now include dataset IDs and sufficient methodological detail for independent interpretation.

Data availability statement confirmed: All GEO accession numbers are listed in accordance with PLOS ONE policy.

────────────────────────────────────────────────────────────

**Summary**

We believe the revisions described above have substantially strengthened the manuscript. We are grateful for the opportunity to improve our work and hope that the revised version now meets the standards of PLOS ONE. We remain available to address any further questions.

Sincerely,

The Authors
