# Supplementary material for: Unraveling the molecular Nexus of Alzheimer’s Disease and HIV encephalitis: The role of cellular senescence and transcriptional regulation
Source: PLoS One. 2026 Aug 13;21(8):e0355548. doi: 10.1371/journal.pone.0355548 (PMC13472368; doi:10.1371/journal.pone.0355548)
Supplement: Peer review history

# Peer review: Decision Letter 0

**DOI:** 10.1371/journal.pone.0355548.r001

**Authors:** Eliseo A Eugenin

17 Mar 2025

Dear Dr. Lu,

Thank you for submitting your manuscript to PLOS ONE. After careful consideration, we feel that it has merit but does not fully meet PLOS ONE’s publication criteria as it currently stands. Therefore, we invite you to submit a revised version of the manuscript that addresses the points raised during the review process.

==============================

**ACADEMIC EDITOR:**

Dear Dr. Lu:

Thank you for submit your manuscript to PLOsone. The manuscript was reviewed by two experts in the area, and both had significant concerns about data presentation, organization, and interpretation. Please can you answers ALL the suggestions.

Best Regards

Eliseo Eugenin

==============================

Please submit your revised manuscript by May 01 2025 11:59PM. If you will need more time than this to complete your revisions, please reply to this message or contact the journal office at plosone@plos.org. When you're ready to submit your revision, log on to https://www.editorialmanager.com/pone/ and select the 'Submissions Needing Revision' folder to locate your manuscript file.

- A rebuttal letter that responds to each point raised by the academic editor and reviewer(s). You should upload this letter as a separate file labeled 'Response to Reviewers'.
- A marked-up copy of your manuscript that highlights changes made to the original version. You should upload this as a separate file labeled 'Revised Manuscript with Track Changes'.
- An unmarked version of your revised paper without tracked changes. You should upload this as a separate file labeled 'Manuscript'.

If you would like to make changes to your financial disclosure, please include your updated statement in your cover letter. Guidelines for resubmitting your figure files are available below the reviewer comments at the end of this letter.

If applicable, we recommend that you deposit your laboratory protocols in protocols.io to enhance the reproducibility of your results. Protocols.io assigns your protocol its own identifier (DOI) so that it can be cited independently in the future. For instructions see: https://journals.plos.org/plosone/s/submission-guidelines#loc-laboratory-protocols. Additionally, PLOS ONE offers an option for publishing peer-reviewed Lab Protocol articles, which describe protocols hosted on protocols.io. Read more information on sharing protocols at https://plos.org/protocols?utm_medium=editorial-email&utm_source=authorletters&utm_campaign=protocols.

We look forward to receiving your revised manuscript.

Kind regards,

Eliseo A Eugenin, Ph.D.

Academic Editor

PLOS ONE

Journal requirements:

When submitting your revision, we need you to address these additional requirements.

1.  Please ensure that your manuscript meets PLOS ONE's style requirements, including those for file naming. The PLOS ONE style templates can be found at

https://journals.plos.org/plosone/s/file?id=wjVg/PLOSOne_formatting_sample_main_body.pdf  and

https://journals.plos.org/plosone/s/file?id=ba62/PLOSOne_formatting_sample_title_authors_affiliations.pdf

2. Please note that PLOS ONE has specific guidelines on code sharing for submissions in which author-generated code underpins the findings in the manuscript. In these cases, we expect all author-generated code to be made available without restrictions upon publication of the work. Please review our guidelines at https://journals.plos.org/plosone/s/materials-and-software-sharing#loc-sharing-code and ensure that your code is shared in a way that follows best practice and facilitates reproducibility and reuse.

3. Thank you for stating the following financial disclosure:

“This research was funded by the Wuxi Science and Technology Development Fund (Grant number: Y20232005).”

Please state what role the funders took in the study.  If the funders had no role, please state: "The funders had no role in study design, data collection and analysis, decision to publish, or preparation of the manuscript."

If this statement is not correct you must amend it as needed.

Please include this amended Role of Funder statement in your cover letter; we will change the online submission form on your behalf.

4. Your ethics statement should only appear in the Methods section of your manuscript. If your ethics statement is written in any section besides the Methods, please move it to the Methods section and delete it from any other section. Please ensure that your ethics statement is included in your manuscript, as the ethics statement entered into the online submission form will not be published alongside your manuscript.

Additional Editor Comments:

Dear Dr. Lu:

Thank you for submit your manuscript to PLOsone. The manuscript was reviewed by two experts in the area, and both had significant concerns about data presentation, organization, and interpretation. Please can you answers ALL the suggestions.

Best Regards

Eliseo Eugenin

[Note: HTML markup is below. Please do not edit.]

Reviewers' comments:

Reviewer's Responses to Questions

**Comments to the Author**

1. Is the manuscript technically sound, and do the data support the conclusions?

Reviewer #1: Partly

Reviewer #2: Partly

**********

2. Has the statistical analysis been performed appropriately and rigorously?

Reviewer #1: Yes

Reviewer #2: I Don't Know

**********

3. Have the authors made all data underlying the findings in their manuscript fully available?

The PLOS Data policy

Reviewer #1: Yes

Reviewer #2: Yes

**********

4. Is the manuscript presented in an intelligible fashion and written in standard English?

Reviewer #1: Yes

Reviewer #2: Yes

**********

Reviewer #1: This study investigates transcriptional regulation involving ZNF148, FOXO3, HDAC1, and ID4 using bioinformatic approaches focusing on possible common mechanism between Alzheimer's disease (AD) and HIV encephalitis (HIVE). Key findings include some key dysregulated genes, unique senescence-associated gene expression profiles in glial cells during HIV infection. Further, authors proposed docking studies to determine a small molecule as therapeutic intervention targeting ZNF148. While the study is interesting and seems to propose novel leading pathways for future therapies, there is lack of literature supports or in vitro studies that supports the bioinformatic approaches. Further, there are several points that should be addressed prior the manuscript being considered acceptable for publication as reported below:

Line 57: citation does not support the sentence. The cited paper talks more generally about HAND not HIVE.

Lines 95-102: this paragraph should be moved to results section, including the figure.

line 106. remove extra space after parentheses.

Figure 1: It is not clear why only two panels have the reference letter. All plots should have a letter, and legends updated accordingly to ensure clarity.

Line 118: please, include the relative references of the datasets used.

line 124: reference to the data set is missing.

Line 144: supporting reference is missing

Line 153: reference for the data set is missing

Line 181: reference for GSE48350 data set is missing. Authors should explain the rational of conducting analysis on different data sets. According to the source, data set is collected using human samples. It is not clear what is the model Samylicus. Authors should report references and more details regarding the experimental approach using this model.

Line 184: authors should verify these numbers and report the source.

Line 208: authors should explain why they did not also include GSE37263 in this analysis.

Line 216: It does not clear the rational behind “minimum of three samples”. Authors should clarify why they did not just use all data available, what power analysis is supporting this statement, and in the case of using a sub-selection of data available, what exclusion criteria was applied and why.

Line 233: the supporting reference for GSE157829 should be cited.

General comment: a table with all data sets used for the study, the type of analysis, the supporting reference to access the dataset, the number of cases analyzed in each data set should be included to improve clarity for the reader.

Lines 320-322: should be considered as discussions not results.

Line 424-441: these kinds of speculations are more suitable for the discussions. Supporting literature should be included.

Line 442: Authors should focus on the description of the data presented in Figure 3 and move any speculation to the discussions.

Line 492: Is not clear what model is the Samylicus. Authors should use a widely accepted nomenclature to make it accessible to the general audience. It is not clear what sample size and what type of experimental approach was used to test this model. Was it a published data set? If so, please, include references. If the tissue was processed in the laboratory, methods of tissue preparation should be reported.

Line 750: Authors should provide an in vitro validation of the docking analysis to be able to refer to “molecular basis of their findings” as docking is not indicative of a functional readout as an electrostatic interaction might not trigger a downstream effect.

Reviewer #2: Zhang et al. utilized bioinformatics tools in this manuscript to identify common differentially expressed genes (DEGs) between Alzheimer's Disease (AD) and HIV Encephalitis (HIVE) with the aim of discovering potential therapeutic targets. However, the manuscript presents significant challenges in readability, requiring considerable effort to navigate through numerous datasets. The authors frequently switch between datasets, making it difficult to follow the analysis. Additionally, the manuscript's organization and flow require substantial revisions to enhance clarity and coherence. By implementing the following revisions, the manuscript can be significantly improved in terms of clarity, readability, and overall organization.

1. Introduction: While the introduction effectively outlines the study's objectives and highlights the importance of the experimental approach, it lacks a discussion on the rationale behind the selection of the tested parameters. Furthermore, it does not provide an explanation of what these parameters are or their relevance to AD and HIVE. To improve clarity and depth, the introduction should be revised to include these essential details.

2. Figure Legends: The legend for Figure 1 should be relocated to the end of the manuscript along with the other figure legends, rather than being included in the introduction.

3. Materials and Methods: This section is currently written in a manner that resembles a results section. It should focus solely on describing the methodologies and technologies used without presenting results or referencing tables.

4. Line 142: The word "respectively" is either unnecessary or, if it refers to the two bioinformatics packages used—each applied to a specific disease—it should be repositioned at the end of the sentence for clarity.

5. Datasets: The manuscript references five datasets across various sections of the Materials and Methods. To enhance clarity, all datasets should be consolidated under a dedicated subsection within this section.

6. Figure 2: It is unclear why certain datasets analyzed in the study, such as GSE48350, are not included in panels A–C. The authors should provide justification for this omission.

7. Data Interpretation: In Figure 2A, FOXO3, HDAC1, and ID4 are downregulated, while ZNF148 is upregulated. However, Figure 2F and 4A-B shows the opposite pattern. Similarly, in Figures 2B and 2E, FOXO3 and ID4 are the only significantly different genes in 2E, whereas all four genes are upregulated in 2B and 3A-D. These discrepancies should be addressed with a clear explanation.

8. Figure Legends Placement: All figure legends should be grouped together after the references rather than being dispersed throughout different results sections.

9. Which data set was used for Fig 3? and what does the bar at the top of PDCDILG2 refer to in 3K?

10. Across different figures, it is a good practice to keep the same colors for the same groups. For example, green is for Ctr in Fig 2 while it represents AD in Fig 5.

11. Fig 7A needs to return to original form and not stretched.

**********

what does this mean?). If published, this will include your full peer review and any attached files.

If you choose “no”, your identity will remain anonymous but your review may still be made public.

**Do you want your identity to be public for this peer review?**  For information about this choice, including consent withdrawal, please see our Privacy Policy

Reviewer #1: No

Reviewer #2: No

**********

[NOTE: If reviewer comments were submitted as an attachment file, they will be attached to this email and accessible via the submission site. Please log into your account, locate the manuscript record, and check for the action link "View Attachments". If this link does not appear, there are no attachment files.]

While revising your submission, please upload your figure files to the Preflight Analysis and Conversion Engine (PACE) digital diagnostic tool, https://pacev2.apexcovantage.com/. PACE helps ensure that figures meet PLOS requirements. To use PACE, you must first register as a user. Registration is free. Then, login and navigate to the UPLOAD tab, where you will find detailed instructions on how to use the tool. If you encounter any issues or have any questions when using PACE, please email PLOS at figures@plos.org. Please note that Supporting Information files do not need this step.

# Author comment: Author response to Decision Letter 1

**DOI:** 10.1371/journal.pone.0355548.r002

20 Aug 2025

Dear Editors and Reviewers,

We sincerely apologize for the delayed revision of our manuscript. We fully appreciate the valuable time and effort you have dedicated to reviewing our work, and we have carefully addressed all your insightful comments and suggestions. The extensive revisions were conducted to ensure the highest quality of presentation, reflecting our deep respect for your expertise and guidance. We are truly grateful for the opportunity to improve our manuscript and for the generous time provided for revisions.

_________________________

### Response to Reviewer #1

Thank you for your thoughtful review and constructive feedback, which have been instrumental in strengthening our manuscript. Your recognition of the study’s potential and detailed suggestions have guided our revisions effectively.

**Comment 1: Line 57 – The citation does not support the sentence, as the cited paper focuses on HAND rather than HIVE.**

**Response 1:** We appreciate this important observation. To address this, we have standardized the terminology throughout the revised manuscript by revising all references to "HIV encephalitis" to "HIV-associated neurocognitive disorders (HAND)" to align with the cited literature, ensuring the citation now appropriately supports the content.

**Comment 2: Lines 95–102 – This paragraph, including the figure, should be moved to the Results section.**

**Response 2:** Thank you for this organizational suggestion. We have relocated the paragraph describing the study workflow to the end of the revised manuscript, following the References, where all figure legends are now consolidated. This improves the logical flow by presenting the methodological overview within the results framework.

**Comment 3: Line 106 – Remove the extra space after the parentheses.**

**Response 3:** We appreciate catching this formatting error. We have carefully reviewed the entire revised manuscript and corrected all instances of extra spaces after parentheses to ensure consistent formatting.

**Comment 4: Figure 1 – Only two panels have reference letters; all plots should have letters, and legends should be updated for clarity.**

**Response 4:** Thank you for highlighting this inconsistency. We have revised Figure 1 in the revised manuscript by adding reference letters to all subpanels and updated the figure legend to clearly correspond with each panel, enhancing readability and clarity.

**Comment 5: Line 118 – Include references for the datasets used.**

**Response 5:** We agree with this suggestion. We have added the appropriate references for all datasets mentioned at their first occurrence in the revised manuscript to ensure traceability and reproducibility.

**Comment 6: Line 124 – Missing reference to the dataset.**

**Response 6:** Thank you for noting this omission. We have added the reference for the dataset at its first mention in the revised manuscript, ensuring all data sources are properly documented.

**Comment 7: Line 144 – Missing supporting reference.**

**Response 7:** We appreciate this reminder. A supporting reference for the bioinformatic method described has been added in the revised manuscript to validate the analytical approach.

**Comment 8: Line 153 – Missing reference for the dataset.**

**Response 8:** To address this, we have included the reference for the dataset at its first mention in the revised manuscript, ensuring all data sources are fully documented.

**Comment 9: Line 181 – Missing reference for GSE48350; explain the rationale for analyzing different datasets and clarify the "Samylicus" model with references and experimental details.**

**Response 9:** Thank you for this comprehensive comment. We have added the reference for GSE48350 at its first mention in the revised manuscript. Additionally, we have clarified the rationale for using multiple datasets in Lines 292–294 of the revised manuscript: different datasets were employed to enable internal validation (using primary datasets) and external validation (using independent cohorts), strengthening the robustness of our findings. The ambiguous term "Samylicus model" has been removed, and we have provided detailed descriptions of the experimental design of GSE48350, including sample characteristics and tissue sources, with corresponding references in the Methods section.

**Comment 10: Line 184 – Verify these numbers and report the source.**

**Response 10:** We have verified the numbers and added a clear citation to the original dataset publication as their source in the revised manuscript, ensuring transparency.

**Comment 11: Line 208 – Explain why GSE37263 was not included in this analysis.**

**Response 11:** We appreciate the need for clarification. GSE37263 was used as an internal dataset for initial discovery analyses. To validate our findings independently, we excluded it from subsequent validation steps in the revised manuscript and instead used external datasets. This approach ensures the reliability of our conclusions through cross-cohort verification.

**Comment 12: Line 216 – Clarify the rationale for "minimum of three samples," including power analysis, exclusion criteria, or why not all data were used.**

**Response 12:** Thank you for this important question. Upon re-evaluation, we recognized that using the full dataset (rather than a subset) would enhance statistical power. Thus, in the revised manuscript, we have analyzed the entire GSE48350 dataset, and the mention of "minimum of three samples" has been removed.

**Comment 13: Line 233 – Cite the supporting reference for GSE157829.**

**Response 13:** After careful revision, we decided to remove the single-cell analysis component from the revised manuscript to streamline focus. Consequently, GSE157829 is no longer used, and this reference is no longer necessary.

**Comment 14: General comment – Include a table summarizing all datasets, analysis types, references, and sample sizes for clarity.**

**Response 14:** We fully agree with this suggestion to enhance transparency. In the revised manuscript, we have integrated a comprehensive overview of all datasets into Figure 1 , which visually presents dataset identifiers, disease types, and tissue source. Additionally, detailed descriptions of each dataset, including references and sample counts, are provided in the Methods section at their first mention.

**Comment 15: Lines 320–322 – These should be in the Discussion, not Results.**

**Response 15:** Thank you for this structural feedback. We have revised the Results section in the revised manuscript to focus solely on presenting findings from the machine learning analysis of key genes, removing speculative interpretations. Contextual discussions have been relocated to the Discussion section.

**Comment 16: Lines 424–441 – These speculations are more suitable for the Discussion and require supporting literature.**

**Response 16:** To address this, we have removed the immune infiltration analysis section from the revised manuscript to refine focus. In the remaining Results sections, we have ensured that only empirical findings are presented, with all interpretive content moved to the Discussion section and supported by relevant literature.

**Comment 17: Line 442 – Focus on describing Figure 3 data and move speculation to the Discussion.**

**Response 17:** We appreciate this guidance. The immune checkpoint analysis (previously Figure 3) has been removed from the revised manuscript. For all remaining results, we have strictly focused on data description in the Results section, with interpretations reserved for the Discussion.

**Comment 18: Line 492 – Clarify the "Samylicus" model with standard nomenclature, sample size, methods, or references; if unpublished, describe tissue preparation.**

**Response 18:** As noted earlier, the ambiguous term "Samylicus model" has been removed from the revised manuscript. All datasets used are publicly available and properly cited, with detailed descriptions of sample characteristics and experimental methods provided in the Methods section.

**Comment 19: Line 750 – Provide in vitro validation of docking analysis, as docking alone does not demonstrate functional effects.**

**Response 19:** We acknowledge this limitation. To ensure the manuscript focuses on well-supported findings, we have removed the molecular docking analysis section from the revised manuscript, as in vitro validation was not available to confirm functional relevance.

_________________________

### Response to Reviewer #2

Thank you for your detailed review and invaluable suggestions to improve the manuscript’s clarity and organization. Your insights on structure and readability have significantly enhanced our presentation.

**Comment 1: Introduction – Lack of rationale for selected parameters, their definitions, and relevance to AD/HIVE; revise to include these details.**

**Response 1:** We agree with the need to strengthen this section. In the revised Introduction, we have added a comprehensive discussion explaining the rationale for focusing on transcriptional regulators and cellular senescence, including their known roles in AD pathogenesis, HAND mechanisms, and shared neuroinflammatory pathways. This provides clear context for their selection and relevance.

**Comment 2: Figure 1 legend – Relocate to the end with other figure legends, not in the Introduction.**

**Response 2:** Thank you for this organizational suggestion. The legend for Figure 1 has been moved to the end of the revised manuscript, following the References, where all figure legends are now consolidated.

**Comment 3: Materials and Methods – Written like a Results section; focus solely on methodologies without presenting results or referencing tables.**

**Response 3:** We have thoroughly revised the Materials and Methods section in the revised manuscript to remove all results-related content and table references. It now exclusively describes analytical pipelines, software tools, and dataset characteristics, adhering to methodological reporting standards.

**Comment 4: Line 142 – "Respectively" is unnecessary or should be repositioned if referring to bioinformatics packages for specific diseases.**

**Response 4:** We appreciate catching this ambiguity. The term "respectively" has been removed, and the sentence has been rephrased for clarity in the revised manuscript, specifying the application of each bioinformatics tool without redundant wording.

**Comment 5: Datasets – Reference five datasets across Materials and Methods; consolidate under a dedicated subsection.**

**Response 5:** This is an excellent suggestion. In the revised manuscript, datasets are introduced in a dedicated subsection within Materials and Methods, providing a centralized overview. Additionally, a visual summary of all datasets (including identifiers, disease types, and tissue sources) is presented in Figure 1, with detailed descriptions and references provided in the Methods section at their first mention.

**Comment 6: Figure 2 – Justify why datasets like GSE48350 are not included in panels A–C.**

**Response 6:** We have clarified this in the revised manuscript. Panels A–C of Figure 2 use internal datasets (GSE37263, GSE3489) for initial discovery of differentially expressed genes. GSE48350 was reserved for external validation, presented in Figure 4, to independently confirm these findings. This separation ensures robust validation of discovery results.

**Comment 7: Data interpretation – Discrepancies in gene expression patterns between figures (e.g., Figure 2A vs. 2F/4A-B; Figure 2B/2E vs. 3A-D); explain these differences.**

**Response 7:** Thank you for highlighting these inconsistencies. To resolve this, we have simplified Figure 2 in the revised manuscript by retaining only volcano plots (removing bean plots) to present differential expression results uniformly. All gene expression patterns are now consistent across figures, with clear labels indicating dataset sources to avoid confusion.

**Comment 8: Figure legends placement – Group all after References, not dispersed in Results.**

**Response 8:** We have implemented this suggestion. All figure legends are now consolidated at the end of the revised manuscript, following the References, for improved readability.

**Comment 9: Figure 3 – Specify the dataset used and explain the bar at the top of PDCDILG2 in 3K.**

**Response 9:** The immune checkpoint analysis (previously Figure 3K) has been removed from the revised manuscript to streamline focus. For remaining figures, dataset sources are clearly indicated in both figure legends and corresponding Results text.

**Comment 10: Consistent colors for groups across figures (e.g., green for Ctrl in Fig 2 vs. AD in Fig 5).**

**Response 10:** We appreciate this attention to detail. In the revised manuscript, we have standardized color coding across all figures, enhancing visual clarity.

**Comment 11: Figure 7A – Restore original proportions, not stretched.**

**Response 11:** As noted in Response 19 to Reviewer #1, the molecular docking analysis (previously Figure 7) has been removed from the revised manuscript, making this adjustment unnecessary.

_________________________

### Additional Modifications and Clarifications

In addition to addressing the reviewers’ comments, we have made the following important revisions:

1. **Author Affiliation Update**: Due to a change in workplace, the first author Hao Zhang’s affiliation has been updated to: *Geriatrics Center, Wuxi Second Geriatric Hospital, Wuxi, Jiangsu Province, China* (previously listed with two affiliations).

2.**Funding Statement**: We have updated the funding disclosure in the revised manuscript to include all supporting grants and clarified the funders’ role:

"This study was supported by the “Wuxi Science and Technology Development Fund (Award Number: Y20232005)”, “2025 Yangzhou University Provincial Key Lab Open Project for Integrative Chinese-Western Geriatric Disease Prevention & Treatment (Award Number: 202528)”, and “2025 Wuxi Aging Research Project (Award Number: WXLN25-A-24)” – all awarded to Hao Zhang. These funds enabled the research: the Wuxi Science and Technology Development Fund provided infrastructure support, including computational resources and database access. The Yangzhou University project funded molecular mechanism exploration, supporting gene expression and pathway analyses. The Wuxi Aging Research Project contributed to diagnostic model development and validation, supporting computational resources for optimization. "

3.**Code Sharing**: In accordance with PLOS ONE guidelines, we have added a statement in the Data Availability section of the revised manuscript indicating that all data underlying this study are fully available without restrictions.

4. **Regarding Figure Submission and PACE Access Issue**: We attempted to access the PACE website (https://pacev2.apexcovantage.com/) to verify figure formatting but encountered technical difficulties accessing the platform. However, we have carefully revised and optimized all figures in the revised manuscript to ensure they meet PLOS ONE’s style requirements, including consistent formatting, resolution, and labeling. We are happy to make further adjustments if needed.

_________________________

We have submitted two versions of the revised manuscript: a clean version and a marked-up version with changes highlighted for your convenience.

Once again, we express our deepest gratitude to both reviewers and editors for your invaluable feedback, patience, and guidance. Your expertise has significantly improved the quality and rigor of our work. We hope the revised manuscript now meets PLOS ONE’s publication criteria.

Thank you for your time and consideration.

Best regards,

Wei Lu

Attachment Submitted filename: Response to Reviewers.docx

# Peer review: Decision Letter 1

**DOI:** 10.1371/journal.pone.0355548.r003

**Authors:** Eliseo A Eugenin

1 Oct 2025

Dear Dr. Lu,

Thank you for submitting your manuscript to PLOS ONE. After careful consideration, we feel that it has merit but does not fully meet PLOS ONE’s publication criteria as it currently stands. Therefore, we invite you to submit a revised version of the manuscript that addresses the points raised during the review process.

==============================

Hi Dr. Lu

Thnak you for resubmitting your manuscript to PLOSone. Both reviewers still have significant and relevant issues with the manuscript that I have to agree with. The answers to the comments of the previous reviewers were insufficient. Please, if you can address the comments, resubmit, because a lack of compliance could result in rejection.

Best Regards

Eliseo Eugenin

==============================

Please submit your revised manuscript by Nov 15 2025 11:59PM. If you will need more time than this to complete your revisions, please reply to this message or contact the journal office at plosone@plos.org. When you're ready to submit your revision, log on to https://www.editorialmanager.com/pone/ and select the 'Submissions Needing Revision' folder to locate your manuscript file.

- A rebuttal letter that responds to each point raised by the academic editor and reviewer(s). You should upload this letter as a separate file labeled 'Response to Reviewers'.
- A marked-up copy of your manuscript that highlights changes made to the original version. You should upload this as a separate file labeled 'Revised Manuscript with Track Changes'.
- An unmarked version of your revised paper without tracked changes. You should upload this as a separate file labeled 'Manuscript'.

If you would like to make changes to your financial disclosure, please include your updated statement in your cover letter. Guidelines for resubmitting your figure files are available below the reviewer comments at the end of this letter.

If applicable, we recommend that you deposit your laboratory protocols in protocols.io to enhance the reproducibility of your results. Protocols.io assigns your protocol its own identifier (DOI) so that it can be cited independently in the future. For instructions see: https://journals.plos.org/plosone/s/submission-guidelines#loc-laboratory-protocols. Additionally, PLOS ONE offers an option for publishing peer-reviewed Lab Protocol articles, which describe protocols hosted on protocols.io. Read more information on sharing protocols at https://plos.org/protocols?utm_medium=editorial-email&utm_source=authorletters&utm_campaign=protocols.

We look forward to receiving your revised manuscript.

Kind regards,

Eliseo A Eugenin, Ph.D.

Academic Editor

PLOS ONE

Journal Requirements:

If the reviewer comments include a recommendation to cite specific previously published works, please review and evaluate these publications to determine whether they are relevant and should be cited. There is no requirement to cite these works unless the editor has indicated otherwise.

Additional Editor Comments:

Hi Dr. Lu

Thnak you for resubmitting your manuscript to PLOSone. Both reviewers still has significant and relevant issue with the manuscript that I have to agreed with. The answers to the comments of the previous reviewers were insufficient. Please, if you can address the comments, resubmit, becasue lack of compliance could result in rejection.

Best Regards

Eliseo Eugenin

[Note: HTML markup is below. Please do not edit.]

Reviewers' comments:

Reviewer's Responses to Questions

**Comments to the Author**

Reviewer #1: All comments have been addressed

Reviewer #3: (No Response)

**********

2. Is the manuscript technically sound, and do the data support the conclusions?

Reviewer #1: Partly

Reviewer #3: Partly

**********

3. Has the statistical analysis been performed appropriately and rigorously?

Reviewer #1: Yes

Reviewer #3: I Don't Know

**********

4. Have the authors made all data underlying the findings in their manuscript fully available?

The PLOS Data policy

Reviewer #1: Yes

Reviewer #3: Yes

**********

5. Is the manuscript presented in an intelligible fashion and written in standard English?

Reviewer #1: Yes

Reviewer #3: Yes

**********

Reviewer #1: Authors addressed most of all my comments. Specifically, regarding comment 19:

"Comment 19: Line 750 – Provide in vitro validation of docking analysis, as docking

alone does not demonstrate functional effects.**

**Response 19:** We acknowledge this limitation. To ensure the manuscript focuses

on well-supported findings, we have removed the molecular docking analysis section

from the revised manuscript, as in vitro validation was not available to confirm

functional relevance"

Authors decided to remove the data given the impossibility of supporting the docking studies with in vitro validation. However, it should be considered that a manuscript that relay only on bioinformatics still has important limitations as validation of data produced using informatics tools should be still validated.

Further, I still have a few comments:

- Figure 1 should be cited in text, I could not find the reference.

- Acronyms should be spelled out first time introduced (ie. AUC at line 22 should be spelled out not passed line 80)

- Line 237: for the clarity of the reader, I would consider refer to "pathway" and not "terms".

Overall, despite much improved, methods are still very descriptive in a like "results/discussions" format. In my opinion, to have a more concise method section would much improve the readability of the article.

Reviewer #3: Zhang H. and co-authors presented a DEG and transcriptional analysis in addition to co-expression analysis to identify diagnostic factors for AD and HIV-HAND using multiple data sets, some are internal data sets and some are publicly available sets for confirmation of their results. They also utilized multiple machine learning models.

The manuscript needs a lot of attention to read smoothly as the use of many supplementary tables and datasets resources make it less cohesive and less reader-friendly. The following are the major points to address:

1. Data sets in data review show all patients info but the reader has to click on each code to get information about the patient gender, age, and diagnosis. Only one data set shows the information in the labeling of the codes (GSE48350). Therefore, it would be easier if either all labels of all sets provide info as done in GSE48350, or a table is presented in each data set with the patients ages, gender and diagnosis.

2. Most of the patients in all data sets are males except maybe one female in some control groups. I wonder if this was chosen or the public data sets are presented as such. This presentation should be mentioned as a limitation of the study that it does not address gender differences. Also, these data then apply only to males so future studies should include females.

3. AD stage of patients is not listed under any data set. It is important to know if the results apply to early or late stage AD cases.

4. My main concern is the very small sample size for all data sets per group. For humans, you cannot provide a solid conclusion from just 6 patients per group.

5. Number of patients or controls in each data set need to be provided under materials and methods for each data set.

6. why does the data set in some data reviews have the summary and materials and methods sections.

7. It would be a good idea to provide a table with all data sets and the number of patients in each set and the % of males and females in that set, plus the different diagnosis categories.

8. All figures need their font to be increased.

9. Arrows in Figure 1 need to be shortened so that they don't cover the headline of the following boxes.

**********

what does this mean?). If published, this will include your full peer review and any attached files.

If you choose “no”, your identity will remain anonymous but your review may still be made public.

**Do you want your identity to be public for this peer review?**  For information about this choice, including consent withdrawal, please see our Privacy Policy

Reviewer #1: No

Reviewer #3: **Yes:** Rabab Al-Lahham

**********

[NOTE: If reviewer comments were submitted as an attachment file, they will be attached to this email and accessible via the submission site. Please log into your account, locate the manuscript record, and check for the action link "View Attachments". If this link does not appear, there are no attachment files.]

While revising your submission, please upload your figure files to the Preflight Analysis and Conversion Engine (PACE) digital diagnostic tool, https://pacev2.apexcovantage.com/. PACE helps ensure that figures meet PLOS requirements. To use PACE, you must first register as a user. Registration is free. Then, login and navigate to the UPLOAD tab, where you will find detailed instructions on how to use the tool. If you encounter any issues or have any questions when using PACE, please email PLOS at figures@plos.org. Please note that Supporting Information files do not need this step.

# Author comment: Author response to Decision Letter 2

**DOI:** 10.1371/journal.pone.0355548.r004

13 Apr 2026

Response to Reviewers

Manuscript PONE-D-25-09126R1

Wei Lu, M.D., on behalf of all authors

2026-04-14

1. Response to Reviewers

Manuscript Number: PONE-D-25-09126R1

Title: Unraveling the Molecular Nexus of Alzheimer’s Disease and HIV Encephalitis: The Role of Cellular Senescence and Transcriptional Regulation

Corresponding Author: Wei Lu, M.D.

Submission Date: April 14, 2026

1.1 Cover Letter to the Academic Editor

Eliseo A. Eugenin, Ph.D.

Academic Editor

PLOS ONE

Date: April 14, 2026

Dear Dr. Eugenin,

We wish to express our deepest and most sincere gratitude to you and the reviewers for the extraordinary time, effort, and expertise dedicated to reviewing our manuscript. We are profoundly grateful for the insightful, constructive, and invaluable comments provided by both reviewers, which have substantially enhanced the quality, clarity, and scientific rigor of our work. Your patience and understanding during our extended revision period are deeply appreciated, and we are truly thankful for the opportunity to improve our manuscript.

We would like to respectfully explain the reasons for our delayed resubmission:

1.Substantial Work Reconstruction: During this revision period, we conducted extensive additional analyses and substantially restructured the manuscript to address all reviewer concerns comprehensively. This included:

oCreating comprehensive summary tables for all datasets with detailed demographic and clinical information

oAdding detailed discussions on study limitations, including gender imbalance, sample size constraints, and the bioinformatics-only nature of our study

oReorganizing the Methods section for improved clarity and conciseness

oRegenerating all figures (Figures 1-8) with enhanced readability and larger font sizes

oSystematically verifying all statistical data and ensuring 100% accuracy

2.Personal Circumstances: The first author experienced a change in institutional affiliation and encountered unforeseen health challenges during the revision process, which unfortunately delayed our progress. We sincerely apologize for any inconvenience this may have caused.

3.Authorship Update: We respectfully request to add Dr. GuoXun Shi as a co-first corresponding author. Dr. Shi made substantial and indispensable contributions to the revised manuscript, particularly in data analysis, interpretation, and methodological innovations. All authors have unanimously agreed to this authorship change, and we are fully prepared to provide any necessary documentation to support this request.

We have carefully and thoughtfully addressed each and every comment raised by the reviewers and the academic editor. Below, please find our detailed point-by-point responses to all comments. All changes in the revised manuscript have been highlighted in the “Revised Manuscript with Track Changes” file for your convenience.

Once again, we extend our heartfelt thanks for your continued consideration, guidance, and support throughout this revision process. We are hopeful that the revised manuscript now meets PLOS ONE’s high publication standards.

With our warmest regards and deepest appreciation,

Wei Lu, M.D.

On behalf of all authors

Geriatrics Center, Wuxi Second Geriatric Hospital

Wuxi, Jiangsu Province, China

Email: luwei5166@gmail.com

1.2 Response to Academic Editor

Editor’s Comment: “Both reviewers still have significant and relevant issues with the manuscript that I have to agree with. The answers to the comments of the previous reviewers were insufficient. Please, if you can address the comments, resubmit, because a lack of compliance could result in rejection.”

Response: We sincerely and deeply appreciate your frank assessment and invaluable guidance. We have taken all reviewer comments with the utmost seriousness and have made comprehensive, thoughtful revisions to address every single concern. We genuinely believe that the revised manuscript now fully meets PLOS ONE’s publication criteria. The specific changes are detailed in our responses to each reviewer below. We are profoundly grateful for your patience and constructive feedback throughout this process.

1.3 Response to Reviewer #1

We wish to express our sincere gratitude to Reviewer #1 for the positive acknowledgment that “Authors addressed most of all my comments” and for recognizing our dedicated efforts in addressing previous concerns. We deeply appreciate the continued constructive and insightful feedback, which has been instrumental in improving our manuscript.

1.3.1 Comment 1: Figure 1 Citation

Reviewer’s Comment: “Figure 1 should be cited in text, I could not find the reference.”

Response: We sincerely apologize for this oversight. We have now added a clear and explicit citation of Figure 1 in the Methods section.

Changes Made: - Added citation: “Analysis workflow is illustrated in Fig. 1.” - Location: Methods section, subsection “Study Design and Data Availability”

1.3.2 Comment 2: Acronym Definitions

Reviewer’s Comment: “Acronyms should be spelled out first time introduced (ie. AUC at line 22 should be spelled out not passed line 80)”

Response: We have carefully and thoroughly reviewed the entire manuscript and ensured that all acronyms are properly defined at their first occurrence.

Changes Made: - Abstract: “area under the curve [AUC]” now defined at first use - FOXO3 diagnostic accuracy values added: “FOXO3 achieved AUC of 0.922 in AD temporal cortex and 0.771 in HAND frontal cortex” - FOXO3-ACE correlation added: “ACE showed strong positive correlation with FOXO3 (R = 0.685)” - Location: Abstract and throughout the manuscript

1.3.3 Comment 3: Terminology Clarity

Reviewer’s Comment: “Line 237: for the clarity of the reader, I would consider refer to ‘pathway’ and not ‘terms’.”

Response: We fully agree with this excellent suggestion for improved clarity. We have replaced “terms” with “pathways” where appropriate throughout the manuscript.

Changes Made: - Changed “Notable terms included” to “Notable pathways included” - Location: Results section, subsection “FOXO3 and ACE as Potential Biomarkers in AD and HAND Pathogenesis”

1.3.4 Comment 4: Methods Section Conciseness

Reviewer’s Comment: “Overall, despite much improved, methods are still very descriptive in a like ‘results/discussions’ format. In my opinion, to have a more concise method section would much improve the readability of the article.”

Response: We have substantially reorganized and condensed the Methods section to improve readability while maintaining essential details for reproducibility. We have also highlighted our key methodological innovations, including the systematic evaluation of 129 ensemble algorithm combinations.

Changes Made: - Removed result-like descriptions from Methods - Streamlined procedural descriptions - Moved interpretative statements to Results/Discussion sections - Emphasized methodological innovation: “We systematically evaluated 129 ensemble algorithm combinations (9 single algorithms, 36 pairwise ensembles, and 84 triple ensembles)” - Location: Entire Methods section has been reorganized

1.3.5 Comment 5: Bioinformatics Limitations Declaration

Reviewer’s Comment: “Manuscripts relying solely on bioinformatics analysis have significant limitations; results require experimental validation”

Response: We fully agree with this critical and important point. We have added comprehensive discussions acknowledging the limitations of bioinformatics-only studies and emphasizing the need for experimental validation.

Changes Made: - Added limitation statement in Discussion section: “Cross-sectional design precludes causal inference regarding temporal relationships. Experimental validation remains necessary to confirm computational predictions. Future studies should incorporate longitudinal designs to establish biomarker dynamics. Additionally, protein-level validation through immunohistochemistry or ELISA would strengthen findings.” - Added limitation statement in Conclusions section: “Future experimental validation will confirm computational predictions. Longitudinal studies should establish biomarker dynamics and causal relationships.” - Emphasized that our findings are computational predictions requiring wet-lab validation - Location: Discussion section (integrated into limitations discussion) and Conclusions section

1.4 Response to Reviewer #3

We wish to express our deepest gratitude to Reviewer #3 (Dr. Rabab Al-Lahham) for the comprehensive, detailed, and highly constructive review. We sincerely appreciate the considerable time and effort invested in providing these invaluable suggestions to improve our manuscript. Your thorough and insightful comments have been extremely helpful in strengthening our work.

1.4.1 Comment 1: Dataset Information Accessibility

Reviewer’s Comment: “Data sets in data review show all patients info but the reader has to click on each code to get information about the patient gender, age, and diagnosis. Only one data set shows the information in the labeling of the codes (GSE48350). Therefore, it would be easier if either all labels of all sets provide info as done in GSE48350, or a table is presented in each data set with the patients ages, gender and diagnosis.”

Response: We completely agree with this excellent suggestion. We have created a comprehensive summary table (Table 1) that consolidates all patient demographic and clinical information for all datasets.

Changes Made: - Created Table 1: “Comprehensive Overview of Alzheimer’s and HIV-Related Neurodegenerative Datasets” - Table includes: GSE ID, Age, Sex, Diagnosis, Stage, N (sample size), Source, Platform, Brain Region, and Year - Location: Results section, after Methods subsection “Study Design and Data Availability”

1.4.2 Comment 2: Gender Imbalance Limitation

Reviewer’s Comment: “Most of the patients in all data sets are males except maybe one female in some control groups. I wonder if this was chosen or the public data sets are presented as such. This presentation should be mentioned as a limitation of the study that it does not address gender differences. Also, these data then apply only to males so future studies should include females.”

Response: This is an excellent and important point. The gender imbalance reflects the characteristics of publicly available neurodegenerative disease cohorts rather than intentional selection. We have added a dedicated paragraph discussing this limitation.

Changes Made: - Added limitation discussion: “All datasets in this study were predominantly male, which reflects the characteristics of public neurodegenerative disease cohorts rather than intentional selection. We did not perform sex-stratified analyses due to insufficient female samples. Consequently, our findings may be primarily applicable to male populations. Future studies should include balanced sex representation to establish generalizability across both sexes.” - Location: Discussion section (integrated into the study limitations discussion)

1.4.3 Comment 3: AD Stage Information

Reviewer’s Comment: “AD stage of patients is not listed under any data set. It is important to know if the results apply to early or late stage AD cases.”

Response: We have added AD stage information to Table 1 for all relevant datasets where this information was available.

Changes Made: - Added “Stage” column to Table 1 showing disease stages (e.g., “AD vs. Ctrl”, “AsymAD vs. AD”, “AD/MCI vs. Ctrl”) - Clarified in Methods which datasets include staging information - Location: Table 1 and Methods section

1.4.4 Comment 4: Small Sample Size Concern

Reviewer’s Comment: “My main concern is the very small sample size for all data sets per group. For humans, you cannot provide a solid conclusion from just 6 patients per group.”

Response: We acknowledge this important limitation. While individual datasets have small sample sizes, our multi-dataset integration approach (HAND n=107, AD n=596) and comprehensive validation across multiple independent cohorts strengthen the statistical power and generalizability of our findings. We have added a discussion of this limitation.

Changes Made: - Added limitation discussion acknowledging small sample sizes in some datasets - Emphasized that multi-dataset integration and cross-validation approaches mitigate this limitation - Location: Discussion section (incorporated into the comprehensive limitations discussion)

1.4.5 Comment 5: Sample Numbers in Methods

Reviewer’s Comment: “Number of patients or controls in each data set need to be provided under materials and methods for each data set.”

Response: We have added detailed sample numbers for each dataset in the Methods section.

Changes Made: - Added specific sample numbers: “For GSE37263 (AD temporal cortex), we initially accessed 16 samples (8 AD, 8 controls) from GEO; after QC assessment… 10 high-quality samples (6 AD, 4 controls) were retained…” - Similar details provided for all datasets - Location: Methods section (incorporated into dataset descriptions throughout)

1.4.6 Comment 6: Redundant Sections

Reviewer’s Comment: “why does the data set in some data reviews have the summary and materials and methods sections.”

Response: We have streamlined the dataset descriptions to eliminate redundancy between Summary and Methods sections.

Changes Made: - Unified format: Summary provides overview, Methods provides specific analytical parameters - Removed redundant information - Location: Throughout Methods section

1.4.7 Comment 7: Summary Table Suggestion

Reviewer’s Comment: “It would be a good idea to provide a table with all data sets and the number of patients in each set and the % of males and females in that set, plus the different diagnosis categories.”

Response: We have implemented this excellent suggestion by creating Table 1, which includes all requested information.

Changes Made: - Table 1 includes: GSE ID, Age, Sex (M/F ratio), Diagnosis categories, Stage, N (total samples), Source, Platform, Brain Region, Year - Location: Results section

1.4.8 Comment 8: Figure Font Size

Reviewer’s Comment: “All figures need their font to be increased.”

Response: We sincerely appreciate this important suggestion. We have carefully reviewed and substantially improved the readability of all figures (Figures 1-8). Font sizes have been enhanced in key figures and panels where clarity could be improved, while maintaining appropriate balance with overall figure layout. We remain fully committed to making any additional adjustments to figure presentation based on editorial guidance.

Changes Made: - Enhanced font sizes in key axis labels, legends, and annotations - Improved overall figure clarity and readability - Maintained consistency across figure panels - Location: All figures (Figures 1-8) - improvements include: - Figure 1: Workflow diagram labels and arrows optimized - Figure 5: Venn map annotations improved - Figure 7: Boxplot axis titles and statistical annotations clarified - Other figures: Layout and clarity optimizations as needed

We welcome any specific suggestions for further improvements.

1.4.9 Comment 9: Figure 1 Arrow Design

Reviewer’s Comment: “Arrows in Figure 1 need to be shortened so that they don’t cover the headline of the following boxes.”

Response: We have redesigned Figure 1 with shortened arrows that no longer obscure text.

Changes Made: - Shortened all connecting arrows - Repositioned arrows to avoid text overlap - Location: Figure 1

1.5 Summary of Major Revisions

1.Table 1: Created comprehensive dataset summary table with demographic and clinical information

2.Figure Improvements: Enhanced readability across all figures with selective font size increases in key panels; redesigned Figure 1 arrows. Committed to further optimization based on editorial feedback.

3.Limitations Discussion: Added comprehensive discussion of study limitations including:

oGender imbalance (predominantly male cohorts)

oSmall sample sizes in some datasets

oBioinformatics-only nature requiring experimental validation

oCross-sectional design limitations

4.Methods Reorganization: Streamlined Methods section for improved readability

5.Acronym Definitions: Ensured all acronyms are defined at first use

6.Figure Citations: Added missing Figure 1 citation in text

7.Authorship: Added Dr. GuoXun Shi as co-fir

Attachment Submitted filename: Response_to_Reviewers.docx

# Peer review: Decision Letter 2

**DOI:** 10.1371/journal.pone.0355548.r005

**Authors:** Eliseo A Eugenin

26 May 2026

Dear Dr. Lu,

Thank you for submitting your manuscript to PLOS ONE. After careful consideration, we feel that it has merit but does not fully meet PLOS ONE’s publication criteria as it currently stands. Therefore, we invite you to submit a revised version of the manuscript that addresses the points raised during the review process.

Please submit your revised manuscript by Jul 10 2026 11:59PM. If you will need more time than this to complete your revisions, please reply to this message or contact the journal office at plosone@plos.org. When you're ready to submit your revision, log on to https://www.editorialmanager.com/pone/ and select the 'Submissions Needing Revision' folder to locate your manuscript file.

- A letter that responds to each point raised by the academic editor and reviewer(s). You should upload this letter as a separate file labeled 'Response to Reviewers'.
- A marked-up copy of your manuscript that highlights changes made to the original version. You should upload this as a separate file labeled 'Revised Manuscript with Track Changes'.
- An unmarked version of your revised paper without tracked changes. You should upload this as a separate file labeled 'Manuscript'.

If you would like to make changes to your financial disclosure, please include your updated statement in your cover letter. Guidelines for resubmitting your figure files are available below the reviewer comments at the end of this letter.

If applicable, we recommend that you deposit your laboratory protocols in protocols.io to enhance the reproducibility of your results. Protocols.io assigns your protocol its own identifier (DOI) so that it can be cited independently in the future. For instructions see: https://journals.plos.org/plosone/s/submission-guidelines#loc-laboratory-protocols. Additionally, PLOS ONE offers an option for publishing peer-reviewed Lab Protocol articles, which describe protocols hosted on protocols.io. Read more information on sharing protocols at https://plos.org/protocols?utm_medium=editorial-email&utm_source=authorletters&utm_campaign=protocols.

As the corresponding author, your ORCID iD is verified in the submission system and will appear in the published article. PLOS supports the use of ORCID, and we encourage all coauthors to register for an ORCID iD and use it as well. Please encourage your coauthors to verify their ORCID iD within the submission system before final acceptance, as unverified ORCID iDs will not appear in the published article. *Only*  the individual author can complete the verification step; PLOS staff *cannot*  verify ORCID iDs on behalf of authors.

We look forward to receiving your revised manuscript.

Kind regards,

Eliseo A Eugenin, Ph.D.

Academic Editor

PLOS One

Journal Requirements:

If the reviewer comments include a recommendation to cite specific previously published works, please review and evaluate these publications to determine whether they are relevant and should be cited. There is no requirement to cite these works unless the editor has indicated otherwise.

Please review your reference list to ensure that it is complete and correct. If you have cited papers that have been retracted, please include the rationale for doing so in the manuscript text, or remove these references and replace them with relevant current references. Any changes to the reference list should be mentioned in the rebuttal letter that accompanies your revised manuscript. If you need to cite a retracted article, indicate the article’s retracted status in the References list and also include a citation and full reference for the retraction notice.

Additional Editor Comments:

Dear Dr. Lu

Thank you for submitting your manuscript to PLOSone. Please correct the comments indicated by the reviewers

Best Regards

Eliseo Eugenin

[Note: HTML markup is below. Please do not edit.]

Reviewers' comments:

Reviewer's Responses to Questions

**Comments to the Author**

Reviewer #1: All comments have been addressed

Reviewer #3: All comments have been addressed

**********

2. Is the manuscript technically sound, and do the data support the conclusions?

Reviewer #1: Yes

Reviewer #3: Partly

**********

3. Has the statistical analysis been performed appropriately and rigorously?

Reviewer #1: Yes

Reviewer #3: Yes

**********

4. Have the authors made all data underlying the findings in their manuscript fully available?

The PLOS Data policy

Reviewer #1: Yes

Reviewer #3: Yes

**********

5. Is the manuscript presented in an intelligible fashion and written in standard English?

Reviewer #1: Yes

Reviewer #3: Yes

**********

Reviewer #1: the manuscript is significantly improved. I only have minor comments:

Comment 1. Figure legends should contain more information. Figure are very dense and Figure legends should allow figures to be self explanatory. For example: Figure 1 legend should briefly describe the workflow.

Comment 2. Panels F and H look stretched. Quality should be fixed.

Reviewer #3: Authors have addressed my previous comments. However, few minor comments need addressing as well as some corrections to be done.

1. Page 5: Table 1:

• age as elderly needs to be changed with at least an age range (78.5 ± 9.2 years for AD temporal cortex, 52.3 ± 8.7 for HAND frontal cortex and 74.2 ± 7.8 for AD blood) like how was described above or elderly needs to be defined in the Table legend.

• Write the number of males and females rather than M/F or the % of males.

• N should be the finalized number used in the study not the initial one chosen, the original numbers can be explained under the method section. If N at the top of the table is for the initial selection before the QC assessment, it needs to be defined as such

• Stay consistent with the number of patients and subjects involved, use the same description, so the number per group needs to be specified rather than a total number for all cases in that data set. For example,

GSE3489 17 AIDS pts (HIVE+ vs. HIVE-)

GSE35864 24 subj (Ctrl, HIV, HAD, HIVE) needs to be specified with the number per group and remove the word subj. Same here GSE48350 AD vs. Ctrl and GSE63060 AD, MCI vs. Ctrl AD/MCI vs. Ctrl

What is HAD in data set GSE35864? It should be HAND

• GSE48350 20-99 yrs is a huge range.

• Again, AD stage for many datasets is not listed

2. Figure 2C; this was done for data set GSE3489, should it be HAND?

3. Figure 2: the reader can get lost and has to go back and forth to check to which data set the analysis in the different figures is done. It is better to either write in the legend again or at the top of each figure the data set number the analysis was performed on. Same for all figures

4. Figures 3E and 4J show no difference in ACE between controls and HAND or AD groups. Then how would it be considered a biomarker for HAND and AD?

5. Result section: Key Genes FOXO3 and ACE Identified in GSE48350 with Diagnostic Potential in GSE122063 Datasets: all data in Figure 4 were for the set GSE48350 which is a hippocampus data set, then how can the different results be applied for cortex (frontal and temporal) and why the data set GSE122063 included in this result section title without being referred to.

**********

what does this mean?). If published, this will include your full peer review and any attached files.

If you choose “no”, your identity will remain anonymous but your review may still be made public.

**Do you want your identity to be public for this peer review?**  For information about this choice, including consent withdrawal, please see our Privacy Policy

Reviewer #1: **Yes:** Claudia Marino

Reviewer #3: No

**********

[NOTE: If reviewer comments were submitted as an attachment file, they will be attached to this email and accessible via the submission site. Please log into your account, locate the manuscript record, and check for the action link "View Attachments". If this link does not appear, there are no attachment files.]

To ensure your figures meet our technical requirements, please review our figure guidelines: https://journals.plos.org/plosone/s/figures

You may also use PLOS’s free figure tool, NAAS, to help you prepare publication quality figures: https://journals.plos.org/plosone/s/figures#loc-tools-for-figure-preparation.

NAAS will assess whether your figures meet our technical requirements by comparing each figure against our figure specifications.

# Author comment: Author response to Decision Letter 3

**DOI:** 10.1371/journal.pone.0355548.r006

14 Jul 2026

Response to Reviewers

Manuscript Number: PONE-D-25-09126R2

Title: Unraveling the Molecular Nexus of Alzheimer's Disease and HIV Encephalitis: The Role of Cellular Senescence and Transcriptional Regulation

────────────────────────────────────────────────────────────

Dear Editor and Reviewers,

We sincerely thank the Editor and both Reviewers for their thoughtful and constructive comments on our revised manuscript. These suggestions have helped us further improve the clarity, accuracy, and quality of our work. We have carefully addressed each point and made corresponding revisions to the manuscript. Below, we provide a point-by-point response detailing all changes made. All references to sections and figures correspond to the revised manuscript with tracked changes.

────────────────────────────────────────────────────────────

Reviewer #1

Comment R1-1: Figure legends should contain more information

Reviewer Comment: "Figure legends should contain more information. Figures are very dense and Figure legends should allow figures to be self explanatory. For example: Figure 1 legend should briefly describe the workflow."

Response: We thank the Reviewer for this valuable suggestion. We fully agree that figure legends should be sufficiently detailed to allow independent interpretation. We have revised all figure legends as follows:

Fig. 1: We have added a concise workflow description summarizing the sequential analytical steps (data acquisition, preprocessing, differential expression, machine learning, transcription factor inference, ensemble evaluation, and blood-based validation), with dataset annotations for each step.

Fig. 2–8: Each figure legend now includes: (i) the dataset accession number(s) used; (ii) a brief description of what each panel represents; (iii) the statistical test applied where relevant.

Specific additions: Fig. 2 now explicitly states which panels correspond to GSE37263 (AD) vs. GSE3489 (HAND). Fig. 4 distinguishes GSE48350 panels (A–E, J) from GSE122063 panels (F–I). Fig. 3, 5, 7, and 8 legends now include dataset identifiers.

(Revised Manuscript: Figure Legends section)

────────────────────────────────────────────────────────────

Comment R1-2: Panels F and H look stretched

Reviewer Comment: "Panels F and H look stretched. Quality should be fixed."

Response: We thank the Reviewer for identifying this quality issue. Upon inspection, we confirmed that Fig. 7 Panels F and H (nomograms for HAND and AD cohorts) were affected by incorrect aspect ratio during figure compilation. We have:

Re-exported both panels from source data at 600 DPI with correct aspect ratio.

Conducted a full quality check on all figure panels to confirm no similar issues exist elsewhere.

All figures will be submitted as high-resolution TIFF files meeting PLOS ONE requirements (≥300 DPI).

(Revised Manuscript: Figure 7; Figure files will be updated in the final submission)

────────────────────────────────────────────────────────────

Reviewer #3

Comment R3-1a: Table 1 — "Elderly" should be replaced with specific age range

Reviewer Comment: "Page 5, Table 1: 'age as elderly' needs to be changed with at least an age range (78.5 ± 9.2 years for AD temporal cortex, 52.3 ± 8.7 for HAND frontal cortex and 74.2 ± 7.8 for AD blood) like how was described above or elderly needs to be defined in the Table legend."

Response: We agree that "Elderly" is insufficiently specific. We have revised Table 1 as follows:

Replaced the "Age" column entries with the specific values already reported in the Methods section: GSE37263 (78.5 ± 9.2 years), GSE3489 (52.3 ± 8.7 years, verified from original manuscript Methods section, now in the table with a footnote marker[§]), GSE63060 (74.2 ± 7.8 years), GSE48350 (20–99 years, verified from the series matrix sample annotations).

For datasets where individual-level age data were not available in GEO records (GSE28160, GSE35864, GSE118553, GSE122063, GSE132903), we have marked the entry as "N/S" (Not Specified) with a footnote.

Added a table footnote explaining the source of age data and acknowledging this limitation.

Limitation acknowledged: We recognize that age information is unavailable for several datasets. This reflects a limitation of publicly available transcriptomic datasets rather than an oversight in our analysis. We have noted this in both Table 1 and the Discussion section.

(Revised Manuscript: Table 1, with revised "Age" column and additional footnotes)

────────────────────────────────────────────────────────────

Comment R3-1b: Table 1 — "M/F" should be replaced with specific numbers of males and females

Reviewer Comment: "Write the number of males and females rather than M/F or the % of males."

Response: We thank the Reviewer for this specification. Upon further inspection of the original GEO records, we found that per-sample sex data were not uniformly available across all datasets. We have revised Table 1 as follows:

GSE63060: 191M / 138F (58% male, from 329 samples) — sex data available from the AddNeuroMed cohort.

GSE48350: 124M / 129F (verified from the series matrix sample annotations, 253 samples across 4 brain regions).

For all other datasets (GSE3489, GSE28160, GSE35864, GSE37263, GSE118553, GSE122063, GSE132903), per-sample sex data were not accessible in the original GEO records. The entry is marked "N/S" (Not Specified) with a footnote.

Correction note: In the previous revision, we reported GSE37263 as 7M/3F based on the subset of 10 post-QC samples. However, upon further verification, we found that per-sample sex information for the 16 original samples (8 AD, 8 Ctrl) was not uniformly recorded in the GEO deposit. To maintain strict data integrity, we have revised this entry to "N/S" in the updated Table 1.

Limitation acknowledged: We recognize that sex information is unavailable for most datasets. This reflects a common limitation of publicly available transcriptomic datasets, particularly postmortem brain tissue studies where demographic annotation is often incomplete. We have noted this in both Table 1 and the Discussion section.

(Revised Manuscript: Table 1, "Sex (M/F)" column updated)

────────────────────────────────────────────────────────────

Comment R3-1c: Table 1 — N should be the final QC number

Reviewer Comment: "N should be the finalized number used in the study not the initial one chosen, the original numbers can be explained under the method section. If N at the top of the table is for the initial selection before the QC assessment, it needs to be defined as such."

Response: We agree. We have revised Table 1 to include two columns: "Total N (final)" for samples included after QC (as specified in Methods), and "Initial N" for the original cohort size before QC filtering. The Methods section already describes the QC process and sample selection criteria for GSE37263 (16→10), GSE3489 (28→12 selected), and GSE63060 (329 all passed QC). For transparency, initial N values are now explicitly listed alongside final N.

(Revised Manuscript: Table 1, with new "Initial N" column; Methods section)

────────────────────────────────────────────────────────────

Comment R3-1d: Table 1 — per-group N should be specified

Reviewer Comment: "The number per group needs to be specified rather than a total number for all cases in that data set. For example, GSE3489 17 AIDS pts (HIVE+ vs. HIVE-); GSE35864 24 subj (Ctrl, HIV, HAD, HIVE) needs to be specified with the number per group and remove the word subj. Same here GSE48350 AD vs. Ctrl and GSE63060 AD, MCI vs. Ctrl AD/MCI vs. Ctrl."

Response: We have revised Table 1 to include per-group sample sizes in the "Diagnosis (per-group N)" column, verified wherever possible from the original GEO SOFT files and series matrix annotations:

GSE3489: HIVE+ (8), HIVE- (6) — verified from SOFT file (14 unique individuals, each with A/B technical replicates; 28 total arrays)

GSE35864: Ctrl (6), HIV (6), HAND (7), HIVE (5) — verified from GEO record (24 subjects across 3 brain regions)

GSE37263: AD (6), Ctrl (4)

GSE63060: AD (145), MCI (80), Ctrl (104)— per-group N from raw GEO data; see footnote for AddNeuroMed classification details

GSE118553: Ctrl (27), AsymAD (33), AD (52)

GSE122063: VaD (8), AD (12), Ctrl (11)

GSE132903: AD (97), Ctrl (98)

GSE48350: AD (28), Ctrl (56) — verified from series matrix titles (84 unique individuals, 253 samples across 4 brain regions)

(Revised Manuscript: Table 1, "Diagnosis (per-group N)" column)

────────────────────────────────────────────────────────────

Comment R3-1e: Table 1 — "HAD" should be "HAND"

Reviewer Comment: "What is HAD in data set GSE35864? It should be HAND."

Response: We apologize for this terminology error. "HAD" (HIV-Associated Dementia) has been corrected to "HAND" (HIV-Associated Neurocognitive Disorders) throughout Table 1 and the entire manuscript, including all figure legends and the main text. We have performed a full-text audit to ensure consistent use of "HAND" throughout.

(Revised Manuscript: Table 1, full-text HAD→HAND replacement)

────────────────────────────────────────────────────────────

Comment R3-1: Table 1 — Data Limitations Statement

Response: We acknowledge that certain datasets (GSE3489, GSE28160, GSE35864) lack complete age, sex, and per-group sample size information in the original GEO records. This reflects the inherent limitations of publicly available transcriptomic datasets rather than an oversight in our analysis. We have noted these data gaps in Table 1 using "N/S" (Not Specified) and have added a comprehensive limitations statement in the Discussion section.

Limitations acknowledged: We recognize that the absence of individual-level demographic data for several HAND datasets limits our ability to perform age- and sex-stratified analyses. This is a common challenge in meta-analyses of public repository data. We have explicitly stated this limitation in the Discussion and recommend that future prospective studies include standardized clinical annotation to enable more granular subgroup analyses.

Table optimization: To balance comprehensiveness with readability, we have streamlined Table 1 from 10 columns to 7 columns by consolidating the "Platform" and "Initial N (Pre-QC)" columns into footnotes. This reduces visual clutter while maintaining all essential information required by the reviewer.

(Revised Manuscript: Table 1, Page 5; Discussion — Limitations section)

────────────────────────────────────────────────────────────

Comment R3-1f: GSE48350 age range 20–99 yrs is a broad range

Reviewer Comment: "GSE48350 20-99 yrs is a huge range."

Response: We acknowledge this concern. GSE48350, obtained from the Harvard Brain Tissue Resource Center, encompasses a wide age range reflecting both normal aging controls and AD cases. We have:

Added a footnote in Table 1 explaining: "GSE48350 spans an age range of 20–99 years, reflecting the heterogeneous donor population. Age-stratified analysis was not feasible due to limited per-stratum sample sizes. This is noted as a limitation."

Expanded the Limitations section in the Discussion to address the potential for age-related confounding.

Note that our primary machine learning models used 5-fold cross-validation, which mitigates overfitting to age-related expression patterns.

(Revised Manuscript: Table 1 footnote; Discussion — Limitations)

────────────────────────────────────────────────────────────

Comment R3-1g: AD stage not listed for many datasets

Reviewer Comment: "Again, AD stage for many datasets is not listed."

Response: We agree that AD stage information is valuable contextual data. We have added a "Disease Stage" column to Table 1. Where available in the original GEO records, staging information has been included (e.g., GSE118553: AsymAD vs. AD). For datasets where staging was not deposited, we have marked "N/S" (Not specified).

Regarding GSE48350 specifically, we note that MMSE scores are available for the AD samples (range 0–27, 80 samples from 28 individuals), but Braak neurofibrillary tangle staging was not deposited in the GEO record. We have therefore retained "N/S" for disease stage in this dataset. This limitation is acknowledged in the Discussion.

(Revised Manuscript: Table 1, new "Disease Stage" column; Discussion — Limitations)

────────────────────────────────────────────────────────────

Comment R3-2: Figure 2C was done for GSE3489 — should it be HAND?

Reviewer Comment: "Figure 2C; this was done for data set GSE3489, should it be HAND?"

Response: The Reviewer is correct. Fig. 2C presents the Venn diagram analysis of overlapping DEGs between AD (GSE37263), HAND (GSE3489), and cellular senescence genes. We have:

Revised the figure legend to explicitly state: "Venn diagram of overlapping DEGs among AD (GSE37263), HAND (GSE3489), and 279 cellular senescence-related genes."

Updated the corresponding Results text to clarify the HAND classification.

Ensured that all dataset-to-condition mappings are consistent throughout.

(Revised Manuscript: Fig. 2C legend; Results section)

────────────────────────────────────────────────────────────

Comment R3-3: Figures should indicate dataset numbers

Reviewer Comment: "Figure 2: the reader can get lost and has to go back and forth to check to which data set the analysis in the different figures is done. It is better to either write in the legend again or at the top of each figure the data set number the analysis was performed on. Same for all figures."

Response: We fully agree. We have systematically annotated all figures with dataset accession numbers:

In each figure legend, the dataset ID is now stated at the beginning (e.g., "Fig. 2. FOXO3 and ACE as potential biomarkers in AD and HAND (GSE37263 for AD panels; GSE3489 for HAND panels).")

In the Results text, each figure citation now includes the dataset ID in parentheses (e.g., "(Fig. 2A, GSE37263)").

Specific dataset assignments for multi-panel figures are clearly delineated (e.g., Fig. 4: GSE48350 for panels A–E, J; GSE122063 for panels F–I).

(Revised Manuscript: All Figure legends; Results section throughout)

────────────────────────────────────────────────────────────

Comment R3-4: ACE shows no difference in Figs. 3E and 4J — how is it a biomarker?

Reviewer Comment: "Figures 3E and 4J show no difference in ACE between controls and HAND or AD groups. Then how would it be considered a biomarker for HAND and AD?"

Response: We thank the Reviewer for this important question, which has prompted us to clarify our analytical framework. We have made the following revisions:

1. Explicit acknowledgment in Results: We have added sentences in the Results section explicitly stating that ACE showed no significant difference between groups in Fig. 3E (HAND) and Fig. 4J (AD).

2. New Discussion paragraph: We have added a dedicated subsection entitled "ACE as a Co-Expression Biomarker Rather Than a Differential Biomarker" in the Discussion. This section explains:

ACE was identified through its strong and consistent co-expression with FOXO3 across multiple independent datasets, not through differential expression analysis.

ACE showed Spearman R = 0.685 (p < 0.01) with FOXO3 in GSE37263 and was consistently ranked as a top feature in regression models predicting FOXO3 expression (Fig. 2Q, 3I, 4D).

ACE functions as a co-expression network biomarker — its value lies in its regulatory association with FOXO3 (implicating renin-angiotensin system involvement) rather than as an independent classifier.

The distinction between differential expression biomarkers and co-expression biomarkers is well established in transcriptomic studies.

In the ensemble diagnostic framework (Fig. 8), ACE alone showed low selection frequency across 129 algorithm combinations (Fig. 8H), consistent with limited independent diagnostic value. The primary diagnostic candidates remain FOXO3, ZNF341, and STAT3.

3. Revised terminology in Results section: We have changed the Results subsection title from "FOXO3 and ACE as Potential Biomarkers in AD and HAND Pathogenesis" to "FOXO3 as a Primary Biomarker and ACE as a Co-Expression Partner in AD and HAND Pathogenesis". This change more accurately reflects ACE's role in our analytical framework.

4. Enhanced conceptual framework: The new Discussion paragraph now includes:

A clear distinction bet

Attachment Submitted filename: Response_to_Reviewers_auresp_3.docx

# Editor report: Decision Letter 3

**DOI:** 10.1371/journal.pone.0355548.r007

**Authors:** Eliseo A Eugenin

23 Jul 2026

<p>Unraveling the Molecular Nexus of Alzheimer's Disease and HIV Encephalitis: The Role of Cellular Senescence and Transcriptional Regulation

PONE-D-25-09126R3

Dear Dr. Lu,

We’re pleased to inform you that your manuscript has been judged scientifically suitable for publication and will be formally accepted for publication once it meets all outstanding technical requirements.

Within one week, you’ll receive an e-mail detailing the required amendments. When these have been addressed, you’ll receive a formal acceptance letter and your manuscript will be scheduled for publication.

An invoice will be generated when your article is formally accepted. Please note, if your institution has a publishing partnership with PLOS and your article meets the relevant criteria, all or part of your publication costs will be covered. Please make sure your user information is up-to-date by logging into Editorial Manager at Editorial Manager® and clicking the ‘Update My Information' link at the top of the page. For questions related to billing, please contact billing support.

If your institution or institutions have a press office, please notify them about your upcoming paper to help maximize its impact. If they’ll be preparing press materials, please inform our press team as soon as possible -- no later than 48 hours after receiving the formal acceptance. Your manuscript will remain under strict press embargo until 2 pm Eastern Time on the date of publication. For more information, please contact onepress@plos.org.

Kind regards,

Eliseo A Eugenin, Ph.D.

Academic Editor

PLOS One

Additional Editor Comments (optional):

Dear Dr. Lu

Thank you for answering the concerns.

Best regards

Eliseo Eugenin

Reviewers' comments:

# Editor report: Acceptance letter

**DOI:** 10.1371/journal.pone.0355548.r008

**Authors:** Eliseo A Eugenin

PONE-D-25-09126R3

PLOS One

Dear Dr. Lu,

I'm pleased to inform you that your manuscript has been deemed suitable for publication in PLOS One. Congratulations! Your manuscript is now being handed over to our production team.

At this stage, our production department will prepare your paper for publication. This includes ensuring the following:

* All references, tables, and figures are properly cited

* All relevant supporting information is included in the manuscript submission,

* There are no issues that prevent the paper from being properly typeset

You will receive further instructions from the production team, including instructions on how to review your proof when it is ready. Please keep in mind that we are working through a large volume of accepted articles, so please give us a few days to review your paper and let you know the next and final steps.

Lastly, if your institution or institutions have a press office, please let them know about your upcoming paper now to help maximize its impact. If they'll be preparing press materials, please inform our press team within the next 48 hours. Your manuscript will remain under strict press embargo until 2 pm Eastern Time on the date of publication. For more information, please contact onepress@plos.org.

You will receive an invoice from PLOS for your publication fee after your manuscript has reached the completed accept phase. If you receive an email requesting payment before acceptance or for any other service, this may be a phishing scheme. Learn how to identify phishing emails and protect your accounts at https://explore.plos.org/phishing.

If we can help with anything else, please email us at customercare@plos.org.

Thank you for submitting your work to PLOS ONE and supporting open access.

Kind regards,

PLOS ONE Editorial Office Staff

on behalf of

Dr. Eliseo A Eugenin

Academic Editor

PLOS One
